# Using patient experiences to evaluate care and expectations in lung cancer: analysis of the English Cancer Patient Experience Survey linked with the national cancer registry

**DOI:** 10.1007/s00520-022-06863-4

**Published:** 2022-02-01

**Authors:** Yvonne Nartey, Laila J. Tata, Aamir Khakwani, Vanessa Beattie, Paul Beckett, Richard B. Hubbard, Iain Stewart

**Affiliations:** 1 Division of Epidemiology and Public Health, University of Nottingham, Nottingham, UK; 2 Aintree University Hospital NHS Foundation Trust, Liverpool, UK; 3 University Hospitals of Derby and Burton NHS Foundation Trust, Derby, UK; 4 Division of Respiratory Medicine, NIHR Biomedical Research Centre, University of Nottingham, Nottingham, UK; 5 National Heart & Lung Institute, Imperial College London, Guy Scadding Building, Cale Street, London, SW3 6LY UK

**Keywords:** Patient experience, England, Lung cancer, Item response, Patient reporting

## Abstract

**Purpose:**

Identification of unmet needs in person centred and supportive care could be limited by differences in experience across specific cancer populations. Using the experiences of people with lung cancer, we assess distinctions according to demographic and clinical characteristics.

**Methods:**

The English Cancer Patient Experience Survey was linked to the national cancer registry. The primary outcome was experience of the lung cancer pathway when assessed in multi-question models developed with item response theory. Secondary outcomes were experience by treatment received and in separate dimensions of the care pathway: up to diagnosis, treatment information, and staff support.

**Results:**

Responses from 15,967 adults with a lung cancer diagnosis between 2009 and 2015 were included. Positive experiences were more likely to be reported by people aged between 65 and 80 (adjusted coefficient 0.08, 95%CI 0.05;0.11), those living in the most deprived areas (adjusted coefficient 0.10, 95%CI 0.05;0.14), diagnosed at lung cancer stage IIA–B (adjusted coefficient 0.09, 95%CI 0.04;0.14), and those diagnosed through inpatient elective admissions (adjusted coefficient 0.17, 95%CI 0.07;0.28). Specific experiences differed across dimensions of care and within lung cancer treatment groups.

**Conclusions:**

Experiences differed according to gender and ethnicity, supporting previous observations in cancer. In contrast to previous studies, people with lung cancer were more likely to report positive pathway experiences at older ages, living in more deprived areas, or diagnosed after stage I, all frequently associated with worse clinical outcomes. The distinct observations in lung cancer specific analyses suggest potential unmet needs, such as in early stage disease and younger age groups.

**Supplementary Information:**

The online version contains supplementary material available at 10.1007/s00520-022-06863-4.

## Introduction

Lung cancer is the most common cancer related cause of death in Europe and mortality rates are higher than any other cancer for both males and females [1]. Whilst survival is a valuable metric for comparisons of outcomes across cancer populations, patient reported outcome measures (PROMs) and patient reported experience measures (PREMs) can offer insights into additional factors important to the individuals, but bespoke tools may be lacking in organ-specific cancer fields [2]. Both PROMs and PREMs are valuable in evidence-based evaluation of services or interventions as they provide outcome information direct from the individual on their functions or feelings without a clinical interpretation, where PREMs support quantification of experience in order to measure whether care quality matches expectations [3].

Cancer patient experience surveys provide an important instrument to assess healthcare quality, as well as opportunities to improve and ensure equality of cancer care [4]. The English Cancer Patient Experience Survey (CPES) has been commissioned as a PREM to report patient feedback since 2009 and has been important in benchmarking cancer care in the English National Health Service (NHS). Studies utilising this valuable source of patient perspective have demonstrated largely positive experiences, as well as discrepancies according to age and gender [5, 6]. However, there exist a number of limitations to previous studies including the pooling of participants with cancers of different organs despite important differences at diagnosis, and a focus upon a single summary question from more than 70 items [5, 6]. Item response theory (IRT) is a method that is gaining popularity in both developing and evaluating person-reported outcomes in patient-centred healthcare. Based on responses to multiple related, discrete questions, associations with the underlying trait can be explored [7–9]

To assess the experiences of people with lung cancer who represent a population with poor prognosis, we apply IRT methodology to CPES responses between 2009 and 2015. We assess whether experience (pre-diagnosis care, information provision, support provided by clinical staff, or combined for overall experience) varied by sociodemographic and clinical characteristics in the entire lung cancer population and when stratified by treatment received, highlighting unmet needs not observed in pooled cancer studies.

## Methods

### Data sources and study population

The study population consisted of people with lung cancer who had National Cancer Registration and Analysis Service (NCRAS) data linked to CPES records. NCRAS is responsible for cancer registration in the National Health Service in England, which provides cancer services free at the point of care. For this study, the NCRAS data consisted of people diagnosed with lung cancer (International Classification of Disease codes version 10: C34*) in English hospitals between 01 January 2009 and 31 December 2015. These data were linked by patient and tumour identifiers with survey results of those who responded to at least one wave of the national CPES questionnaire between 2010 and 2015 (wave 1 to wave 5). Multiple responses across waves made by the same patient were matched on patient-tumour identifiers to ensure that only the patient’s first chronological response was used for analysis. The method for this research including the representativeness of the population has previously been described, which demonstrated greater representation of people who received anti-cancer treatment [10]. Routes to diagnosis as reported by NCRAS were used to define pathway of cancer diagnosis (i.e. GP/primary care referral, emergency presentation, inpatient elective, two-week wait pathway). Individual patient treatment modalities were constructed with Hospital Episode Statistics, consistent with methods used by the English National Lung Cancer Audit (NLCA) [11]: surgery (receipt 1 month before to 6 months after diagnosis), combined chemotherapy and radiotherapy, chemotherapy or radiotherapy alone (receipt 1 month before to 9 months after diagnosis), or no anti-cancer treatment.

### Patient experience outcome measures

CPES was commissioned by the UK Department of Health and the sampling frame included people aged 16 years and older with a primary cancer diagnosis, seen as an inpatient or day case in English NHS hospitals for cancer related treatment over a three-month period each year (varying slightly across annual waves). CPES includes over 70 multiple choice questions; modifications of survey sections made across years were addressed by identifying questions that could be mapped across all five CPES waves to obtain a consistent and complete study population. Multiple waves were ordered according to the item numbering used in wave 5 (Supplementary Document 1; Table S1-S3).

We included all responses, categorising “positive” and “negative” based on the criteria used for reporting annual CPES findings [12, 13], whilst responses indicating uncertainty or neither negative nor positive experience were categorised as “neutral” and included in models using a Likert approach (negative 0, neutral 1, positive 2; Supplementary Table 4). We grouped CPES questions according to four key dimensions of the clinical care pathway experience: pre-diagnosis care, receiving diagnosis and treatment information, clinical staff support, staff support specifically as an inpatient, as well as a combination of the above for an overall experience of the cancer pathway.

### Statistical analysis

Patient reporting can lead to missing responses through non-completion or non-applicability, which leads to listwise exclusion of participants when using traditional regression models. IRT was used to assess latent experience in the four key dimensions of the clinical care pathway as well as overall experience, including all participants regardless of missing question responses. The graded response model was selected and developed with model assumptions verified [14, 15]. Detailed information on item selection is provided as supplementary methods (Supplementary Document 2). A graded response model was constructed with model assumptions achieved; exploratory factor analysis verified selected items as unidimensional and locally independent with one dominant factor. Questions included in final IRT models were specified according to the reliability in distinguishing experience within the key dimensions of the clinical care pathway experience across all waves (Table 1, Tables S1-S3). This approach supported analysis of latent experiences derived from multiple questions, rather than multiple testing of associations on single questions. Theta values represent a measure of the underlying trait derived from the IRT model, scaled to have mean 0 and a standard deviation of 1. Greater theta values indicated a more positive experience whilst a less positive experience was represented by negative theta values; on the original scale this reflected a total possible questionnaire score between 0 and 34 from 17 items rated negatively or positively, respectively (Figure S1).

**Table 1:** Items included in final IRT-specified clinical dimensions of CPES

Clinical dimension	Item number	Item description
1 Experience of pre-diagnosis care	5	Beforehand, did you have all the information you needed about your test?
	7	Were the results of the test explained in a way you could understand?
	9	How do you feel about the way you were told you had cancer?
	11	When you were told you had cancer, were you given written information about the type of cancer you had?
2 Experience of receiving diagnosis and treatment information	13	Were the possible side effects of treatment(s) explained to you in a way you could understand?
	14	Were you offered practical advice and support in dealing with the side effects of your treatment(s)?
	16	Were you involved as much as you wanted to be in decisions about your care and treatment?
	26	After the operation, did a member of staff explain how it had gone in a way you could understand?
	49	Did the doctors or nurses give your family or someone close to you all the information they needed to help care for you at home?
3 Experience of clinical staff support	18	How easy or difficult has it been for you to contact your Clinical Nurse Specialist?
	19	When you have had important questions to ask your Clinical Nurse Specialist, how often have you got answers you could understand?
	20	Did hospital staff give you information about support or self-help groups for people with cancer?
	22	Did hospital staff give you information about how to get financial help or any benefits you might be entitled to?
	41	While you were being treated as an outpatient or day case, did you find someone on the hospital staff to talk to about your worries and fears?
4 Experience of clinical staff support as an inpatient	30	If your family or someone else close to you wanted to talk to a doctor, were they able to?
	35	During your hospital visit, did you find someone on the hospital staff to talk to about your worries and fears?
	36	Did the hospital staff do everything they could to help control your pain?

Items numbered according to CPES wave 5. Overall experience of care pathway based on responses to all clinical dimensions

Linear regression models were specified for overall experience, each of the key dimensions of the clinical care pathway experience, and treatment modality strata, to assess how a person’s sociodemographic and clinical cancer characteristics were associated. Estimates are reported as beta-coefficients (β) with 95% confidence intervals (95%CI) expressed as model theta values relative to a reference group. All variables were initially assessed in a series of univariable analyses, followed by multivariable models to mutually adjust for sociodemographic and clinical characteristics assessed. Adjusted β coefficients are presented. All analyses were performed in Stata 16 (StataCorp, College Station, TX, USA).

## Results

A total of 15,967 people diagnosed with lung cancer responded to the CPES between 2009 and 2015, 83.1% were diagnosed with non-small cell lung cancer, and 15.2% were diagnosed with small cell lung cancer (Table 2). Median age at diagnosis was 68 years (interquartile range 74–62). Most respondents were male (53.6%), 65 to 80 years old (58.8%) and were diagnosed with stage IV disease (30.3%). Individuals who reported their ethnicity as non-white represented 3.5% of respondents and 3.4% of respondents did not receive anti-cancer treatment. Most (45.8%) were diagnosed through the two-week wait pathway, 12.6% by emergency presentation and 1.8% via an inpatient elective route. The number of complete responses to questions included in the models varied between 20.1 and 100%; neutral responses to individual questions varied between 0.7 and 43.9% (Table S4).

**Table 2:** Characteristics of people with lung cancer responding to the cancer patient experience survey waves 1–5 (*N* = 15,967)

	*n*	%
Patient demographic		
Sex		
Male	8561	53.6
Female	7406	46.4
Age (years)		
< 65	5442	34.1
65–80	9394	58.8
> 80	1131	7.1
Ethnicity		
White	15,405	96.5
Non-white	562	3.5
Index multiple deprivation		
1—least deprived	2557	16.0
2	3222	20.2
3	3297	20.7
4	3354	21.0
5—most deprived	3537	22.2
Comorbidity score*		
0	6589	41.3
1	3473	21.8
2–3	2428	15.2
4 +	3477	21.8
Cancer features		
Year of diagnosis		
2009/2010	3280	20.5
2011	3212	20.1
2012	3302	20.7
2013	3028	19.0
2014/2015	3145	19.7
Stage		
Stage IA–IB	2884	18.1
Stage IIA–IIB	1930	12.1
Stage IIIA–IIIB	4547	28.5
Stage IV	4843	30.3
Unknown	1763	11.0
Route of diagnosis		
Emergency presentation	2012	12.6
GP referral	4062	25.4
Inpatient elective	287	1.8
Other outpatient	2161	13.5
TWW	7318	45.8
Missing	127	0.8
Lung cancer type		
Carcinoid	273	1.7
NSCLC	13,268	83.1
SCLC	2426	15.2
Anti-cancer treatment		
Number of treatments		
0	537	3.4
1	7216	45.2
2	7727	48.4
3	487	3.0
Treatment modality		
NSCLC only		
No treatment	496	3.7
Surgery	4945	37.3
Chemo and radio	3750	28.3
Chemotherapy only	2769	20.9
Radiotherapy only	1308	9.9
SCLC only		
No treatment	21	0.9
Surgery	102	4.2
Chemo and radio	1921	79.2
Chemotherapy only	302	12.5
Radiotherapy only	80	3.3

*NSCLC*, non-small cell lung cancer; *SCLC*, small cell lung cancer. Deprivation quintiles presented. *Charlson index of comorbidity presented. *TWW*, two-week wait; *GP*, general practitioner (primary care)

### Associations between people’s characteristics and their overall experience of the lung cancer care pathway

**Fig. 1:**
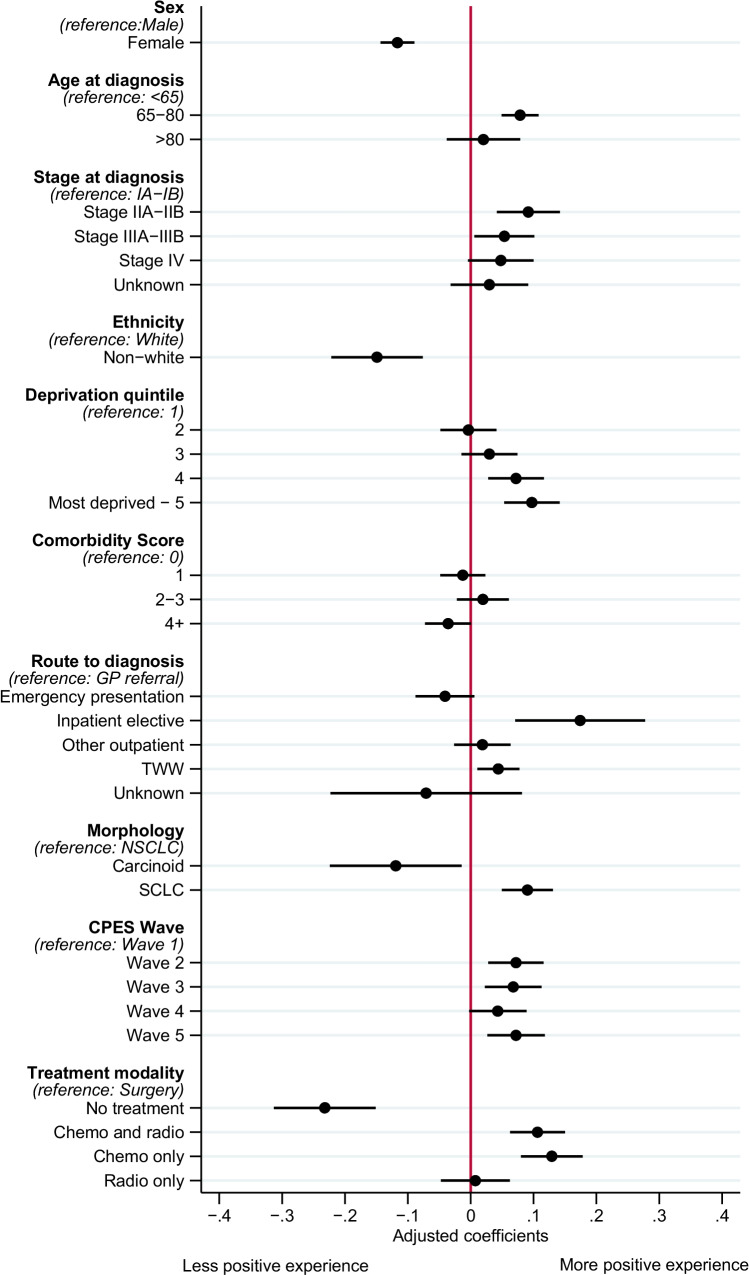
Association of CPES respondent characteristics and their overall experience of the lung cancer care pathway (*N* = 15,967). Multivariable linear regression coefficients adjusted for all variables presented, with 95% confidence interval for overall experience of lung cancer clinical care pathway. Higher estimates indicate more positive experience; lower estimates indicate less positive experience. *N* = 15,967. TWW, two-week wait; NSCLC, non-small cell lung cancer; SCLC, small cell lung cancer

Being female was associated with a less positive overall experience compared to being male (β − 0.12, 95%CI − 0.14; − 0.09) (Fig. 1). Responses from people who reported an ethnicity that was non-white were associated with less positive experiences overall (β − 0.15, 95%CI − 0.23; − 0.07), whilst people between 65 and 80 years old were more likely to report a positive experience compared to younger individuals with lung cancer (β 0.08, 95%CI 0.05;0.11). Being from a more socioeconomically deprived area was associated with a more positive experience of lung cancer care overall (deprivation index 5: β 0.10, 95%CI 0.05;0.14), whilst respondents with stage IIA–B or stage IIIA–B lung cancer were more likely to report a positive experience than stage IA–B (β 0.09, 95%CI 0.04;0.14 and β 0.05, 95%CI 0.00;0.10, respectively).

People diagnosed through the two-week wait route were more likely to report a positive overall experience compared to diagnosis via a GP referral (β 0.04, 95%CI 0.01;0.08), similarly for the 1.8% of the cohort diagnosed via an inpatient elective route (β 0.17, 95%CI 0.07;0.28). A small cell lung cancer diagnosis was associated with a more positive experience compared to non-small cell lung cancer (β 0.09, 95%CI 0.05;0.13), whilst the 1.7% diagnosed with carcinoid lung cancer were more likely to report a less positive experience overall (β − 0.12, 95%CI − 0.22; − 0.02). Compared to people who received surgery, receipt of chemotherapy (with or without radiotherapy) was associated with a more positive experience of the lung cancer care pathway, whilst the 3.5% of CPES respondents who did not receive anti-cancer treatment were more likely to report a less positive experience (β − 0.23, 95%CI − 0.31; − 0.16). Compared to the first CPES wave, respondents from subsequent waves were more likely to report a positive experience.

### Contrasting associations of people’s characteristics with experiences across dimensions of the clinical care pathway

**Fig. 2:**
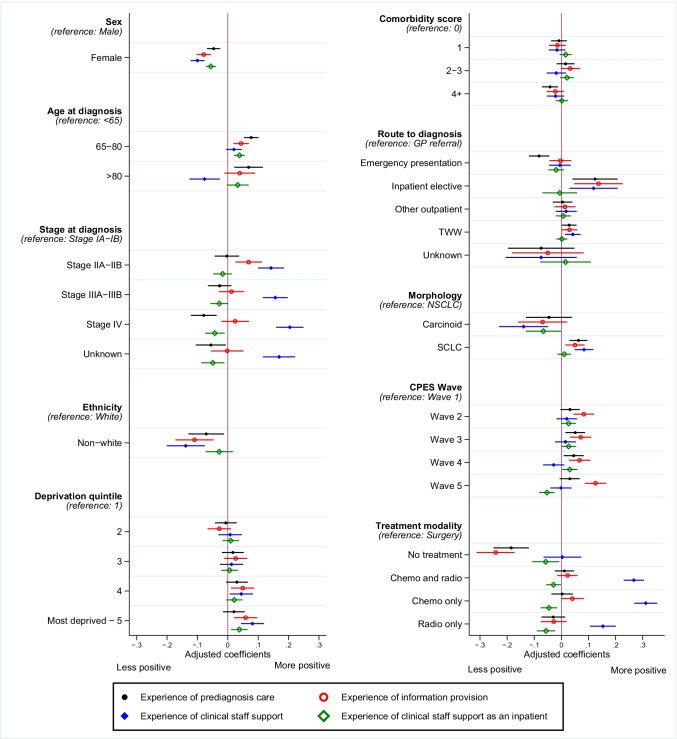
Association of CPES respondent characteristics and their experience of separate dimensions of the care pathway: pre-diagnosis care, information provision, clinical staff support, inpatient experience (*N* = 15,967). Multivariable linear regression coefficients adjusted for mutual confounding and presented with 95% confidence interval according to key survey sections of clinical cancer care pathway: (A) satisfaction up to and including diagnosis; (B) satisfaction with information regarding condition and treatment; (C) experience of NHS staff support across pathway; (D) experience of NHS staff support as an inpatient. Higher estimates indicate more positive experience; negative estimates indicate less positive experience. *N* = 15,967. TWW, two-week wait; NSCLC, non-small cell lung cancer; SCLC, small cell lung cancer

Several associations observed for overall experience were similar within the individual dimensions, including being female, reporting ethnicity other than white, index of multiple deprivation, and lung cancer type (Fig. 2, Table S5).

We also identified personal characteristics that demonstrated discrepancies according to the respective survey dimensions. Compared with individuals aged under 65, those aged over 80 were more likely to report a positive experience up to and including diagnosis, but were a less positive experience regarding experience of NHS staff support across the pathway (β 0.07, 95%CI 0.02;0.11 and β − 0.08, 95%CI − 0.12; − 0.03, respectively). Individuals aged between 65 and 80 were more likely to report a positive experience up to and including diagnosis, regarding information related to their diagnosis and treatment, and regarding staff support specifically as an inpatient, when compared to those aged under 65.

Relative to the experience of individuals with stage IA–IB lung cancer, there was a positive association at stage IIA–IIB regarding the experience of information related to their diagnosis and treatment (β 0.07, 95%CI 0.02;0.11), as well as experience of clinical staff support (β 0.16, 95%CI 0.11;0.20), which was similarly more likely to be positive in more advanced lung cancer stages (stage IV β 0.20, 95%CI 0.16;0.25). People with stage IV or unreported staging of lung cancer were more likely to report a less positive experience of pre-diagnosis care (stage IV β − 0.08, 95%CI − 0.12; − 0.04), as well as a less positive experience of staff support as an inpatient (stage IV β − 0.04, 95%CI − 0.08; − 0.01).

Experiences of staff support as an inpatient did not vary according to route to lung cancer diagnosis, although a more positive experience of clinical staff support overall was associated with the two-week wait route and inpatient elective admissions, relative to GP referral (two-week wait β 0.04, 95%CI 0.01;0.07, inpatient elective β 0.12, 95%CI 0.03;0.21). People diagnosed via an inpatient elective admission were also more likely to report a positive experience of pre-diagnosis care, and regarding information related to their diagnosis and treatment, compared to those diagnosed via GP referral (β 0.12, 95%CI 0.05;0.20 and β 0.14, 95%CI 0.05;0.22, respectively). Diagnosis via an emergency presentation was associated with a less positive experience of pre-diagnosis care (β − 0.08, 95%CI − 0.12; − 0.04), but no associations were observed according to information or staff support. Compared to surgery, receipt of other anti-cancer treatments was associated with a less positive experience of staff support specifically as an inpatient. Conversely, people who received chemotherapy and radiotherapy were more likely to report a positive experience of clinical staff support overall (β 0.27, 95%CI 0.23;0.31).

### Contrasting associations of people’s characteristics with overall experiences according to lung cancer treatment modality

**Fig. 3:**
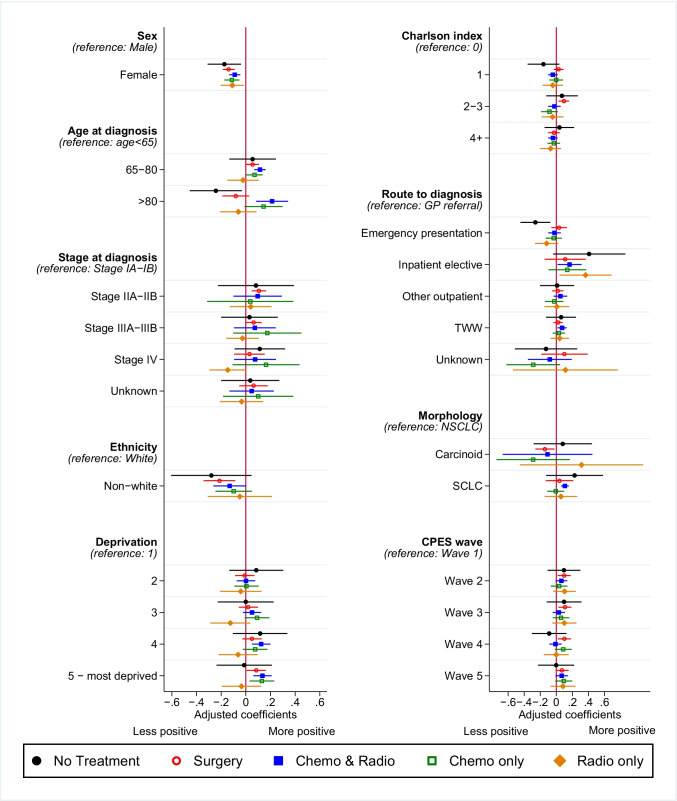
Association of CPES respondent characteristics and their overall experience of the lung cancer pathway, stratified by treatment modality (*N* = 15, 967). Multivariable linear regression coefficients adjusted for all variables presented, with 95% confidence interval for overall experience of lung cancer clinical care pathway, according to the following strata: 1. No receipt of anti-cancer treatment; 2. Receipt of surgery; 3. Receipt of combined chemotherapy and radiotherapy treatment; 4. Receipt of chemotherapy only; 5. Receipt of radiotherapy only. Higher estimates indicate more positive experience; lower estimates indicate less positive experience. *N* = 15,967. TWW, two-week wait; NSCLC, non-small cell lung cancer; SCLC, small cell lung cancer

Consistent with overall experience and within clinical dimensions, being female was associated with more negative experiences compared to male within each treatment modality (Fig. 3, Table S6). Age groupings older than 65 reached significantly more positive experiences in the combined chemotherapy and radiotherapy treatment modality (β 0.11, 95%CI 0.07;0.16), whilst people who received no anti-cancer treatment and were in the over 80 age group reported a more negative experience than those younger (β − 0.24, 95%CI − 0.46; − 0.03). People who were of non-white ethnicity and received surgery or combined chemotherapy and radiotherapy reported more negative experience than those who were white (β − 0.21, 95%CI − 0.34; − 0.09 and β − 0.13, 95%CI − 0.26;0.00, respectively).

Surgery recipients diagnosed with stage IIA–IIB (β 0.11, 95%CI 0.05;0.16) or stage IIIA–IIIB (β 0.06, 95%CI 0.00;0.13), as well as those with 2–3 major comorbidities (β 0.09, 95%CI 0.03;0.16) reported more positive experiences than earlier stage or no comorbidities, respectively. People who received surgery, combined chemotherapy and radiotherapy, or chemotherapy only and were in the most deprived socioeconomic quintile reported more positive experiences than those in the least deprived (β 0.08, 95%CI 0.01;0.16 and β 0.14, 95%CI 0.06;0.21 and β 0.13, 95%CI 0.03;0.23, respectively). People who received combined chemotherapy and radiotherapy, or radiotherapy only, reported a more positive experience when diagnosed through an inpatient elective route than those through a GP referral (β 0.16, 95%CI 0.02;0.31 and β 0.36, 95%CI 0.04;0.69, respectively), whilst people who did not receive anti-cancer therapy reported more negative experience when diagnosed following emergency presentation (β − 0.26, 95%CI − 0.45; − 0.07).

Compared to a NSCLC diagnosis, people who received surgery reported a more negative experience when diagnosed with carcinoid lung cancer subtype (β − 0.14, 95%CI − 0.26; − 0.02), whilst people who received combined chemotherapy and radiotherapy reported more positive experience when diagnosed with SCLC (β 0.11, 95%CI 0.06;0.15).

## Discussion

We report a novel study that uses a large longitudinal sample of CPES respondents with a primary diagnosis of lung cancer and linkages to national cancer registry, adjusting regression models for mutual confounding using all characteristics assessed. These observations demonstrate that for people with lung cancer, positive pathway experiences were more frequently associated with living in a more deprived area and being aged between 65 and 80 years compared to those diagnosed younger. Females were more likely to report less positive experiences, which was similar for people of non-white ethnicity. People diagnosed at stage I appeared to have less positive experiences than those diagnosed with stage IIA–B or stage IIIA–B, and those diagnosed through inpatient elective or two-week wait routes reported more positive experiences than GP referrals. Many findings were consistent in sub-analyses; however, discrepancies were also observed across clinical dimensions and within treatment modalities that highlight possible unmet needs that should be considered when planning patient-centred interventions or developing organ specific cancer PREMs.

### Leveraging cancer specific experiences to identify distinctions

We observe several associations of positive experience in patient groups that commonly have worse relative health outcomes [16], which is in contrast to studies that combine participants with cancers from different organs. Advanced tumour stage at diagnosis and living in areas of high socioeconomic deprivation were strongly associated with lower satisfaction in analysis combining breast, prostate, colon, rectal, and lung cancer types [5]. We observe that people from more deprived areas were more likely to report a positive overall experience, and specifically within dimensions of receiving diagnosis and information on treatment, as well as dimensions of clinical staff support, and across anti-cancer treatments. These distinctions may arise due to differences in outcome classification using a single summary question compared to models that incorporate multiple responses, or the concordance between individual-level deprivation and area-level measures [17]. Education level is a reasonable predictor of individual income and deprivation [17]; our findings are thus consistent with US lung cancer data that demonstrated worse experiences were associated with higher educational attainment [18]. A recent study observed higher patient reported burden in more socio-economically deprived areas [19]; however, as people with lung cancer represented approximately 10% of responses, the representation of cancer type in analysis of PREMs and PROMs should be explored further.

Our analysis of 15,967 people with a primary lung cancer diagnosis over five survey waves demonstrated that more advanced staging is not always associated with lower satisfaction, which may reflect distress regarding pathway or prognostic uncertainties in early stage lung cancer. Positive experiences at later stage diagnoses were most apparent in the dimension of clinical staff support and when stratified by those who received surgery, supporting the role of advanced practice nursing in addressing holistic needs in limited disease [20]. In the small sample of people diagnosed through the elective inpatient route, people were more likely to report more positive experiences compared to GP referral, particularly within treatment modalities that included radiotherapy, which is in contrast to people with colorectal cancer for whom NHS screening is available [21]. A study that included all cancer types represented in CPES observed lower likelihood of positive experiences in people who were diagnosed through inpatient elective routes, as well as for those who were sampled more than 1 year since first treatment [6]. As lung cancer has a 1 year survival rate of 37% in the UK and is frequently diagnosed as advanced disease through emergency presentation, such general experiences are unlikely to reflect those of the lung cancer population.

### Consistencies with previous studies

People in older age categories were more likely to report positive experiences, compared to those aged under 65. This finding was consistent with studies showing less CPES satisfaction in younger age groups across multiple cancer types [6, 22], which may be owing to added domestic, employment, and financial responsibilities [23]. Females reported less positive experiences compared to males across all dimensions modelled and in all treatment modality strata, supporting observations in previous studies [5, 6]. Stable distinctions in pooled and specific cancer populations demonstrate opportunities to understand unmet needs and how perceptions of a cancer diagnosis, as well as possible differences in the subjective interpretation of questions, may be linked to age and sex.

Where people did not report white ethnicity and were not in receipt of anti-cancer treatment, associations with experience were frequently less positive. This may reflect consistency in experiences of small comparator groups relative to large, highlighting the importance of engaging with minority communities and addressing the representation of individuals who are treated palliatively after diagnosis. Studies have shown that early palliation can improve survival outcomes in lung cancer [24], whilst National Institute of Care Excellence (NICE) guidelines in the UK state that individuals with lung cancer who would benefit from supportive or palliative care should be identified and referred to specialist providers early without delay. Bespoke PREMs developed in lung cancer could provide further insights into the experience of supportive care [25], and adapted sampling approaches may reduce biases in representation between CPES lung cancer respondents and the English national lung cancer population [10].

### Strengths and limitations

To our knowledge, this is the first study to model a large sample of lung cancer specific patient experiences using multiple question responses over multiple survey waves, taking advantage of IRT methodology and linkages between the cancer registry and CPES. Our analysis on IRT-developed models enabled all responses to be included, maximising use of patient-reported data by identifying questions which adequately distinguish experience traits in discrete clinical dimensions of the care pathway and overall [14, 15]. We were able to demonstrate distinctions between dimensions across patient characteristics, supporting IRT as a complementary analytical approach to focus decision-making. Future development of nationwide PREMs with IRT could limit demographic response bias, support modelling of non-binary perspectives, gather valuable insights across multiple clinical areas whilst minimising statistical power trade-off from multiple testing, and minimise the survey burden on individuals living with cancer.

This study is limited by discrepancies in CPES comparisons to the national lung cancer population, in particular under representation of people who received radiotherapy or who were not in receipt of anti-cancer treatment, those diagnosed through emergency presentation, stage IV lung cancer, and aged over 80 [10, 11]. There is a likely contribution of sampling and responder bias that favours people with a better prognosis. Nationally, 15% of the UK lung cancer registry is represented by non-white ethnicity [10, 11], whilst this figure was 3.5% of the CPES population, which suggests possible inequities and potential barriers, including language and cultural perceptions [26]. Receipt of treatment within this study refers to the pathway the individual followed according to healthcare data and may not reflect treatment receipt at the time of survey sampling. The majority of questions in CPES offer minimal granularity; we addressed this by defining a three category ordinal Likert scale to be assessed in a graded response IRT model, although patient responses captured across an ordinal scale may offer more sensitivity in the future. Individual values of the latent trait for experience are normally distributed on a scale centred at zero similar to a z-score; however, this is a logistic function of the expected questionnaire score and limits interpretation of the size of effect in the original scale. IRT restricted the number of CPES questions within final models due to strict methodological assumptions, which supports focus upon informative questions where responses vary in the population but may exclude questions considered important by cancer services and users.

### Conclusion

The time and personal views contributed by people with lung cancer allow a direct evaluation of whether user expectations of care are met. CPES presents a vital resource of lung cancer experiences, particularly within the context of poor prognosis where sampling is time-sensitive and burdensome. We highlight that people diagnosed with lung cancer at stage I, aged under 65, or from less deprived socio-economic areas may need additional information and clinical staff support. Experiences of lung cancer care did not meet expectations in females or people from minority communities. We provide quantitative foundations for the development of lung cancer specific PREMs that could help focus responder effort, and we identified potential areas of unmet need in English lung cancer care.

## Supplementary Information

Below is the link to the electronic supplementary material.

Supplementary file1 (PDF 1986 KB)

## Data Availability

The datasets analysed in the current study can be requested from Public Health England.

## Code availability

All analyses were performed using commands available in Stata SE16.0 (StataCorp, TX).
