# Supplementary material for: Using patient experiences to evaluate care and expectations in lung cancer: analysis of the English Cancer Patient Experience Survey linked with the national cancer registry
Source: Support Care Cancer. 2022 Feb 1;30(5):4417–28. doi: 10.1007/s00520-022-06863-4 (PMC8942895; doi:10.1007/s00520-022-06863-4)

National Cancer Patient Experience Survey

This questionnaire is about your care and treatment for cancer. Its purpose is to provide information which can help the NHS monitor and improve the quality of health services for future patients.

Who should complete the questionnaire?
The questions should be answered by the person named in the letter enclosed with this questionnaire. If that person needs help to complete the questionnaire, the answers should be given from their point of view – not the point of view of the person who is helping.

Completing the questionnaire
For each question please tick clearly inside the box that is closest to your views using a black or blue pen. Don't worry if you make a mistake; simply cross out the mistake and put a tick in the correct box. Please **do not** write your name or address anywhere on the questionnaire.

If you would prefer, you may also complete this survey online. To do this, please refer to the login information which can be found in the letter accompanying this survey.

IMPORTANT INFORMATION

To make sure the information we collect is useful, we need to collect some personal details from you and to access information held about you in other NHS databases. The purpose of collecting this information is to generate aggregated statistics about the care and treatment people receive. These statistics will be used to compare the differences in care and treatment by different providers and to understand what may be causing these. The results will be used to measure and improve the quality of healthcare services.

By completing this questionnaire you are giving your consent for the information provided to be used for the above purposes. Specifically, you are agreeing that:

- Your personal details and relevant health information can be held and used by an organisation contracted to NHS England to analyse the data
- Other information about you held by the Patient Demographics Service, the Secondary Users Service and other NHS databases can be held and used by an organisation contracted to NHS England to analyse the data

Your personal information will be handled securely and anonymised after analysis and before any publication.

Your personal information will not be released by NHS England or third party organisations working on its behalf unless required by law or where there is a clear overriding public interest.

You can withdraw the information you give the NHS in this questionnaire upon request, up to the point at which data are analysed and personal details removed.

If you have any queries about the questionnaire,
please call the FREEPHONE helpline number **on 0800 783 1775**

Taking part in this survey is voluntary

Published reports will not contain any personal details

SEEING YOUR GP

These questions are about what happened before you went into hospital about cancer.

1. Before you were told you needed to go to hospital about cancer, how many times did you see your GP (family doctor) about the health problem caused by cancer?

1 ☐ **None** – I went straight to hospital

2 ☐ **None** – I went to hospital following a cancer screening appointment

3 ☐ I saw my GP **once** 1

4 ☐ I saw my GP **twice** 1

5 ☐ I saw my GP **3 or 4** times 0

6 ☐ I saw my GP **5 or more** times 0

7 ☐ Don't know / can't remember

2. How do you feel about the length of time you had to wait before your **first appointment with a hospital doctor**?

1 ☐ I was seen as soon as I thought was necessary 1

2 ☐ I should have been seen a bit sooner 0

3 ☐ I should have been seen a lot sooner 0

3. How long was it from the time you first thought something might be wrong with you until you first saw a GP or other doctor?

1 ☐ Less than 3 months

2 ☐ 3-6 months

3 ☐ 6-12 months

4 ☐ More than 12 months

5 ☐ Don't know / can't remember

DIAGNOSTIC TESTS

4. In the last 12 months, have you had a diagnostic test(s) for cancer such as an endoscopy, biopsy, mammogram, or scan at one of the hospitals named in the covering letter?

1 ☐ Yes **➔ Go to Q5**

2 ☐ No **➔ Go to Q8**

Thinking about the LAST time you had a diagnostic test for your cancer at one of the hospitals named in the covering letter ….

5. Beforehand, did you have all the information you needed about your test? **(Tick ALL that apply)**

1 ☐ Yes

2 ☐ No, I would have liked more **written** Information

3 ☐ No, I would have liked more **verbal** information

4 ☐ I did not need / want any information

5 ☐ Don't know / can't remember

6. Overall, how did you feel about the length of time you had to wait for your test to be done?

1 ☐ It was about right 1

2 ☐ It was a little too long 0

3 ☐ It was much too long 0

4 ☐ Don't know / can't remember

7. Were the results of the test explained in a way you could understand?

1 ☐ Yes, completely 1

2 ☐ Yes, to some extent 0

3 ☐ No, I did not understand the explanation 0

4 ☐ I did not have an explanation but would have liked one 0

5 ☐ I did not need an explanation

6 ☐ Don't know / can't remember

FINDING OUT WHAT WAS WRONG WITH YOU

8. When you were first told that you had cancer, had you been told you could bring a family member or friend with you?

1 ☐ Yes 1
2 ☐ No 0
3 ☐ It was not necessary
4 ☐ I was told by phone or letter
5 ☐ Don't know / can't remember

9. How do you feel about the way you were told you had cancer?

1 ☐ It was done sensitively 1
2 ☐ It should have been done **a bit** more sensitively 0
3 ☐ It should have been done **a lot** more sensitively 0

10. Did you understand the explanation of what was wrong with you?

1 ☐ Yes, I completely understood it 1
2 ☐ Yes, I understood some of it 0
3 ☐ No, I did not understand it 0
4 ☐ Don't know / can't remember

11. When you were told you had cancer, were you given **written** information about the type of cancer you had?

1 ☐ Yes, and it was **easy** to understand 1
2 ☐ Yes, but it was **difficult** to understand 0
3 ☐ No, I was not given written information about the type of cancer I had 0
4 ☐ I did not need written information
5 ☐ Don't know / can't remember

DECIDING THE BEST TREATMENT FOR YOU

12. Before your cancer treatment started, were your treatment options explained to you?

1 ☐ Yes, completely 1
2 ☐ Yes, to some extent 0
3 ☐ No 0
4 ☐ There was only one type of treatment that was suitable for me
5 ☐ Don't know / can't remember

13. Were the possible side effects of treatment(s) explained in a way you could understand?

1 ☐ Yes, definitely 1
2 ☐ Yes, to some extent 0
3 ☐ No, side effects were not explained 0
4 ☐ I did not need an explanation
5 ☐ Don't know / can't remember

14. Were you offered practical advice and support in dealing with the side effects of your treatment(s)?

1 ☐ Yes, definitely 1
2 ☐ Yes, to some extent 0
3 ☐ No, I was not offered any practical advice or support 0
4 ☐ Don't know / can't remember

15. Before you started your treatment(s), were you also told about any side effects of the treatment that could affect you in the future rather than straight away?

1 ☐ Yes, definitely 1

2 ☐ Yes, to some extent 0

3 ☐ No, future side effects were not explained 0

4 ☐ I did not need an explanation

5 ☐ Don't know / can't remember

16. Were you involved as much as you wanted to be in decisions about your care and treatment?

1 ☐ Yes, definitely 1

2 ☐ Yes, to some extent 0

3 ☐ No, but I would like to have been more involved 0

4 ☐ Don't know / can't remember

CLINICAL NURSE SPECIALIST

A Clinical Nurse Specialist is a specialist cancer nurse who makes sure you get the right care and gives you help and advice on coping with cancer.

17. Were you given the name of a Clinical Nurse Specialist who would support you through your treatment?

1 ☐ Yes 1 **➔ Go to Q18**

2 ☐ No 0 **➔ Go to Q20**

3 ☐ Don't know / can't remember **➔ Go to Q20**

18. How easy or difficult has it been for you to contact your Clinical Nurse Specialist?

1 ☐ Very easy 1

2 ☐ Quite easy 1

3 ☐ Neither easy nor difficult 0

4 ☐ Ouite difficult 0

5 ☐ Very difficult 0

6 ☐ I have not tried to contact my Clinical Nurse Specialist

19. When you have had important questions to ask your Clinical Nurse Specialist, how often have you got answers you could understand?

1 ☐ All or most of the time 1

2 ☐ Some of the time 0

3 ☐ Rarely or never 0

4 ☐ I have not asked any questions

SUPPORT FOR PEOPLE WITH CANCER

20. Did hospital staff give you information about support or self-help groups for people with cancer?

1 ☐ Yes **1**
2 ☐ No, but I would have liked information **0**
3 ☐ It was not necessary
4 ☐ Don't know / can't remember

21. Did hospital staff discuss with you or give you information about the impact cancer could have on your day to day activities (for example, your work life or education)?

1 ☐ Yes **1**
2 ☐ No, but I would have liked a discussion or information **0**
3 ☐ It was not necessary / relevant to me
4 ☐ Don't know / can't remember

22. Did hospital staff give you information about how to get financial help or any benefits you might be entitled to?

1 ☐ Yes **1**
2 ☐ No, but I would have liked information **0**
3 ☐ It was not necessary
4 ☐ Don't know / can't remember

23. Did hospital staff tell you that you could get free prescriptions?

1 ☐ Yes **1**
2 ☐ No, but I would have liked information **0**
3 ☐ It was not necessary
4 ☐ Don't know / can't remember

OPERATIONS

24. During the last 12 months, have you **had an operation** (such as removal of a tumour or lump) at one of the hospitals named in the covering letter?

1 ☐ Yes **➔ Go to Q25**
2 ☐ No **➔ Go to Q27**

Thinking about the LAST time you went into one of the hospitals named in the covering letter for an operation for your cancer …

25. Beforehand, did you have all the information you needed about your operation? **(Tick ALL that apply)**

1 ☐ Yes
2 ☐ No, I would have liked more **written** Information
3 ☐ No, I would have liked more **verbal** information
4 ☐ I did not need / want information
5 ☐ Don't know / can't remember

26. After the operation, did a member of staff explain how it had gone in a way you could understand?

1 ☐ Yes, completely **1**
2 ☐ Yes, to some extent **0**
3 ☐ No, but I would have liked an explanation **0**
4 ☐ I did not need an explanation

HOSPITAL CARE AS AN INPATIENT

27. During the last 12 months, have you had an operation or stayed overnight **for cancer care** at one of the hospitals named in the covering letter?

1 ☐ Yes ➔ **Go to Q28**

2 ☐ No ➔ **Go to Q40**

Thinking about the LAST time you had an operation or stayed overnight <u>for cancer care</u> at one of the hospitals named in the covering letter…

28. Did groups of **doctors *and* nurses** talk in front of you as if you weren't there?

1 ☐ Yes, often 0

2 ☐ Yes, sometimes 0

3 ☐ No 1

29. Did you have confidence and trust in the **doctors** treating you?

1 ☐ Yes, in all of them 1

2 ☐ Yes, in some of them 0

3 ☐ No, in none of them 0

30. If your family or someone else close to you wanted to talk to a **doctor**, were they able to?

1 ☐ Yes, definitely 1

2 ☐ Yes, to some extent 0

3 ☐ No 0

4 ☐ No family or friends were involved

5 ☐ My family did not want to talk to a doctor

6 ☐ I did not want my family or friends to talk to a doctor

31. Did you have confidence and trust in the ward **nurses** treating you?

1 ☐ Yes, in all of them 1

2 ☐ Yes, in some of them 0

3 ☐ No, in none of them 0

32. In your opinion, were there enough **nurses** on duty to care for you in hospital?

1 ☐ There were always or nearly always enough on duty 1

2 ☐ There were sometimes enough on duty 0

3 ☐ There were rarely or never enough on duty 0

33. While you were in hospital did the **doctors *and* nurses** ask you what name you prefer to be called by?

1 ☐ Yes, all of them did 1

2 ☐ Only some of them did 0

3 ☐ None of them did 0

34. Were you given enough privacy when discussing your condition or treatment?

1 ☐ Yes, always 1

2 ☐ Yes, sometimes 0

3 ☐ No 0

35. During your hospital visit, did you find someone on the hospital staff to talk to about your worries and fears?

1 ☐ Yes, definitely 1

2 ☐ Yes, to some extent 0

3 ☐ No 0

4 ☐ I had no worries or fears

36. Do you think the hospital staff did everything they could to help control your pain?

1 ☐ Yes, definitely 1

2 ☐ Yes, to some extent 0

3 ☐ No 0

4 ☐ I did not have any pain

37. Overall, did you feel you were treated with respect and dignity while you were in the hospital?

1 ☐ Yes, always 1

2 ☐ Yes, sometimes 0

3 ☐ No 0

38. Were you given clear **written** information about what you should or should not do after leaving hospital?

1 ☐ Yes 1

2 ☐ No 0

3 ☐ Don't know / can't remember

39. Did hospital staff tell you who to contact if you were worried about your condition or treatment after you left hospital?

1 ☐ Yes 1

2 ☐ No 0

3 ☐ Don't know / can't remember

HOSPITAL CARE AS A DAY PATIENT / OUTPATIENT

Thinking about the LAST time you attended hospital as a day patient or outpatient <u>for cancer care</u> at one of the hospitals named in the covering letter…

40. During the last 12 months, have you been treated as an outpatient or day case **for cancer care** at one of the hospitals named in the covering letter?

1 ☐ Yes **➔ Go to Q41**

2 ☐ No **➔ Go to Q49**

41. While you were being treated as an outpatient or day case, did you find someone on the hospital staff to talk to about your worries and fears?

1 ☐ Yes, definitely 1

2 ☐ Yes, to some extent 0

3 ☐ No 0

4 ☐ I did not have any worries or fears

42. The **last** time you had an outpatients appointment with a cancer doctor, did they have the right documents, such as medical notes, x-rays and test results?

1 ☐ Yes 1

2 ☐ No 0

3 ☐ I didn't have an appointment with a cancer doctor

4 ☐ Don't know / can't remember

43. During the last 12 months, have you had radiotherapy at any of the hospitals named in the covering letter?

1 ☐ Yes **➔ Go to Q44**

2 ☐ No **➔ Go to Q46**

44. Beforehand, did you have all of the information you needed about your radiotherapy treatment?

1 ☐ Yes, completely 1

2 ☐ Yes, to some extent 0

3 ☐ No 0

4 ☐ I did not need any information

45. Once you started your treatment, were you given enough information about whether your radiotherapy was working in a way you could understand?

1 ☐ Yes, completely 1
2 ☐ Yes, to some extent 0
3 ☐ No 0
4 ☐ It is too early to know if my radiotherapy is working
5 ☐ I did not need any information

46. During the last 12 months, have you had chemotherapy at any of the hospitals named in the covering letter?

1 ☐ Yes ➔ **Go to Q47**
2 ☐ No ➔ **Go to Q49**

47. Beforehand, did you have all of the information you needed about your chemotherapy treatment?

1 ☐ Yes, completely 1
2 ☐ Yes, to some extent 0
3 ☐ No 0
4 ☐ I did not need any information

48. Once you started your treatment, were you given enough information about whether your chemotherapy was working in a way you could understand?

1 ☐ Yes, completely 1
2 ☐ Yes, to some extent 0
3 ☐ No 0
4 ☐ It is too early to know if my chemotherapy is working
5 ☐ I did not need any information

HOME CARE AND SUPPORT

49. Did the doctors or nurses give your family or someone close to you all the information they needed to help care for you at home?

1 ☐ Yes, definitely 1
2 ☐ Yes, to some extent 0
3 ☐ No 0
4 ☐ No family or friends were involved
5 ☐ My family or friends did not want or need information
6 ☐ I did not want my family or friends to be involved

50. **During** your cancer treatment, were you given enough care and support from health or social services (for example, district nurses, home helps or physiotherapists)?

1 ☐ Yes, definitely 1
2 ☐ Yes, to some extent 0
3 ☐ No 0
4 ☐ I did not need help from health or social services
5 ☐ Don't know / can't remember

51. Once your cancer treatment **finished**, were you given enough care and support from health or social services (for example, district nurses, home helps or physiotherapists)?

1 ☐ Yes, definitely 1
2 ☐ Yes, to some extent 0
3 ☐ No 0
4 ☐ I did not need help from health or social services
5 ☐ I am still having treatment
6 ☐ Don't know / can't remember

CARE FROM YOUR GENERAL PRACTICE

52. As far as you know, was your GP given enough information about your condition and the treatment you had at the hospital?

1 ☐ Yes 1
2 ☐ No 0
3 ☐ Don't know / can't remember

53. Do you think the GPs and nurses at your general practice did everything they could to support you while you were having cancer treatment?

1 ☐ Yes, definitely 1
2 ☐ Yes, to some extent 0
3 ☐ No, they could have done more 0
4 ☐ My general practice was not involved

YOUR OVERALL NHS CARE

54. Did the different people treating and caring for you (such as GP, hospital doctors, hospital nurses, specialist nurses, community nurses) work well together to give you the best possible care?

1 ☐ Yes, always 1
2 ☐ Yes, most of the time 0
3 ☐ Yes, some of the time 0
4 ☐ No, never 0
5 ☐ Don't know / can't remember

55. Have you been given a care plan? *A care plan is a document that sets out your needs and goals for caring for your cancer. It is an agreement or plan between you and your health professional to help meet those goals*

1 ☐ Yes 1
2 ☐ No 0
3 ☐ I do not know / understand what a care plan is
4 ☐ Don't know / can't remember

56. Overall, how would you rate the administration of your care (getting letters at the right time, doctors having the right notes/tests results, etc.)?

1 ☐ Very good 1
2 ☐ Good 1
3 ☐ Neither good nor bad 0
4 ☐ Quite bad 0
5 ☐ Very bad 0
6 ☐ Don't know / can't remember

57. Overall, how do you feel about the length of time you had to wait when attending clinics and appointments for your cancer treatment?

1 ☐ It was much too long 0
2 ☐ It was a little too long 0
3 ☐ It was about right 1
4 ☐ Don't know / can't remember

58. Since your diagnosis, has anyone discussed with you whether you would like to take part in cancer research?

1 ☐ Yes 1
2 ☐ No 0
3 ☐ No, but I would have liked them to 0
4 ☐ Don't know / can't remember

59. Overall, how would you rate your care? (**Please circle a number**)

Very poor — Very good

0	1	2	3	4	5	6	7	8	9	10
0	0	0	0	0	0	0	1	1	1	1
0	1	2	3	4	5	6	7	8	9	10

Will score Q59 two ways – average of 1-10 and % scoring 7 or above

YOUR CONDITION

We would like to understand a little bit more about your cancer and how you are now. Please answer these questions about the cancer that was treated at the hospital(s) named in the covering letter.

60. How long is it since you were first treated for **this cancer**?

1 ☐ Less than 1 year

2 ☐ 1 to 5 years

3 ☐ More than 5 years

4 ☐ Don't know / can't remember

61. Had your cancer spread to other organs or parts of your body at the time you were first told you had cancer?

1 ☐ Yes ➔ **Go to Q63**

2 ☐ No ➔ **Go to Q62**

3 ☐ Don't know ➔ **Go to Q63**

4 ☐ Does not apply to my type of cancer ➔ **Go to Q63**

62. Which of the following applies?

1 ☐ My cancer has been taken out / treated, without any sign of further problem

2 ☐ My cancer was taken out / treated without any sign of further problem, but has since come back / spread to other parts of my body

3 ☐ None of the above options apply to my type of cancer

4 ☐ I would prefer not to say

5 ☐ I don't know

ABOUT YOU

If you are helping someone to complete this questionnaire, please make sure this information is the patient's not your own.

63. What year were you born?

(Please write in) e.g.

1	9	4	4

64. Are you male or female?

1 ☐ Male

2 ☐ Female

65. Which of the following options best describes how you think of yourself?

1 ☐ Heterosexual or Straight

2 ☐ Gay or Lesbian

3 ☐ Bisexual

4 ☐ Other

5 ☐ Prefer not to say

66. Do you have any of the following long-standing conditions? **(Tick ALL that apply)**

1 ☐ Deafness or severe hearing impairment

2 ☐ Blindness or partially sighted

3 ☐ A long-standing physical condition

4 ☐ A learning disability

5 ☐ A mental health condition

6 ☐ A long-standing illness, such as HIV, diabetes, chronic heart disease, or epilepsy

67. Could we send you a survey in the future to ask about your health and healthcare?

1 ☐ Yes, and I understand that this does **NOT** mean that I would have to take part in the future survey

2 ☐ No, I would prefer you not to contact me again

68. Is English your first language?

1 ☐ Yes

2 ☐ No

69. What is your ethnic group? **(Tick ONE only)**

a. WHITE

1 ☐ English / Welsh / Scottish / Northern Irish / British

2 ☐ Irish

3 ☐ Gypsy or Irish Traveller

4 ☐ Any other White background (Please write in box)

b. MIXED / MULTIPLE ETHNIC GROUPS

5 ☐ White and Black Caribbean

6 ☐ White and Black African

7 ☐ White and Asian

8 ☐ Any other Mixed / multiple ethnic background (Please write in box)

c. ASIAN OR ASIAN BRITISH

9 ☐ Indian

10 ☐ Pakistani

11 ☐ Bangladeshi

12 ☐ Chinese

13 ☐ Any other Asian background (Please write in box)

d. BLACK / AFRICAN / CARIBBEAN / BLACK BRITISH

14 ☐ African

15 ☐ Caribbean

16 ☐ Any other Black / African / Caribbean background (Please write in box)

e. OTHER ETHNIC GROUP

17 ☐ Arab

18 ☐ Any other ethnic group (Please write in box)

OTHER COMMENTS

Thinking about the **hospital named in the covering letter**, if there is anything else you would like to tell us about your experience of NHS cancer care, please do so here.

Please note that the comments you provide in the box below will be looked at in full by the NHS Trust, NHS England and researchers analysing the data. We will remove any information that could identify you before publishing any of your feedback.

Was there anything particularly good about your NHS cancer care?

Was there anything that could have been improved?

Any other comments?

Supplementary Document 1

Appended CPES survey 2015

Supplementary Document 2

Supplementary Methods: construction of graded response item response theory models

In item response theory (IRT), an underlying trait is expressed as a continuum on an arbitrary scale (theta value), e.g. satisfaction with care. An average theta value is centred at zero, with affirmation of the latent trait in positive theta and refuting the latent trait in negative theta. IRT predicts a value for the latent trait according to responses to items/questions within a survey or test. Parameters of discrimination (how well an item could differentiate people across the trait) and difficulty (50% probability of endorsing a particular response) are assigned to each item, used to calculate a theta value for each respondent. Four key assumptions are required for a robust IRT model: monotonicity (probability of endorsing a question continuously increases as the latent trait increases), invariance (estimation of the question parameters are independent of the characteristics of the sample), local independence (answers to questions are not dependent on other questions), and unidimensionality (questions within a model measure a single dominant latent trait).

Graded response models (GRM) were constructed for ordered categorical responses: “negative”, “neutral” or “positive” coded as 0, 1 and 2, respectively. CPES methodology was followed for categorising responses “yes, definitely” as 2 (positive), whilst “to some extent” or “no” was categorised as 0 (negative). We also included respondents who were considered neutral (did not need support, didn’t know the answer, or where responses were unknown despite applicability). Where questions were not applicable to respondents, answers were classified as missing; IRT models are designed to handle missing item data without listwise deletion.

For each trait according to the clinical survey section, boundary characteristic curves for categorical response were used to assess discrimination (steep curve, little overlap) and difficulty (value at which 50% endorsed), and along with category characteristic curves, informed item selection

(Supplementary Figure 3). Exploratory factor analysis verified items as unidimensional and locally independent with one dominant factor. Where no dominant factor was initially confirmed, there was a further breakdown and assessment of questions until suitable models were built. The model and question fit of data was confirmed with Akaike's and Bayesian information criterion derived from likelihood-ratio tests. The selected questions were used within the models to derive an individual's level of experience (Theta value) for the latent trait e.g. experience up to diagnosis; a greater value indicates better level of experience.

Assessing differential item functioning is an essential aspect of IRT model building to remove demographic bias that is independent from the clinical context being assessed. In this context we used IRT to test the performance of a survey, rather than development, and we were particularly interested in the differences that may be attributed to a number of respondent characteristics. We did not remove any item that showed differential item functioning.

DRAFT

Supplementary Table 1. Items included in Item Response Theory models of dimension of the clinical care pathway and overall experience

Dimension of cancer care pathway	Clinical survey section/ Latent trait	Questions assessed	Questions in model	Variance from factor analysis(%)
Satisfaction up to and including diagnosis	1	1,2,5,7,8,9,10,11	5,7,9,11	43
Satisfaction of condition & treatment information	2	12,13,14,16,25,26,49	13, 14, 16, 26, 49	49
Experience of NHS staff support across pathway	3	17,18,19,20,22,23,41, 49, 50, 51	18,19, 20, 22, 41	38
Experience of NHS staff support – inpatient only	4	28,29,30,31,32,34,35,36,37, 38, 39	30, 35, 36	53
Overall satisfaction of NHS cancer care	Overall experience of pathway		5,7,9,11, 13, 14, 16, 26, 18,19, 20, 22, 41, 30,35,36, 43, 46, 47	30

DRAFT

Supplementary Table 2. Discrimination and difficulty parameters of items in IRT models.

Item	Item description	discrimination (95%CI)	discrimination * (95%CI)	Coded response	difficulty(95%CI)	Difficulty* (95%CI)
**Clinical dimension 1: Experience up to and including diagnosis**						
5	Beforehand, did you have all the information you needed about your test?	1.96(1.79 ; 2.13)	1.41(1.32 ; 1.51)	>=1	-2.11(-2.21 ; -2.01)	-2.50(-2.62 ; -2.38)
				2	-1.89(-1.98 ; -1.81)	-2.24(-2.34 ; -2.13)
7	Were the results of the test explained in a way you could understand?	1.94(1.78 ; 2.10)	1.50(1.43 ; 1.58)	>=1	-1.09(-1.14 ; -1.04)	-1.22(-1.27 ; -1.17)
				2	-0.95(-1.00 ; -0.91)	-1.06(-1.11 ; -1.02)
9	How do you feel about the way you were told you had cancer?	1.23(1.14 ; 1.31)	0.96(0.90 ; 1.01)	>=1	-1.67(-1.76 ; -1.58)	-1.98(-2.08 ; -1.88)
				2	-1.55(-1.63 ; -1.47)	-1.84(-1.93 ; -1.74)
11	When you were told you had cancer, were you given written information about the type of cancer you had?	1.22(1.14 ; 1.30)	1.06(1.01 ; 1.10)	>=1	-0.86(-0.91 ; -0.81)	-0.94(-0.99 ; -0.89)
				2	-0.15(-0.18 ; -0.12)	-0.16(-0.20 ; -0.12)
**Clinical dimension 2: Experience of condition and treatment information**						
13	Were the possible side effects of treatment(s) explained to you in a way you could understand?	1.96(1.82 ; 2.09)	1.59(1.52 ; 1.66)	>=1	-1.03(-1.07 ; -0.98)	-1.13(-1.18 ; -1.09)
				2	-0.78(-0.81 ; -0.74)	-0.86(-0.89 ; -0.82)
14	Were you offered practical advice and support in dealing with the side effects of your treatment(s)?	2.86(2.46 ; 3.27)	2.19(2.00 ; 2.39)	>=1	-0.61(-0.66 ; -0.56)	-0.70(-0.75 ; -0.64)
				2	-0.42(-0.47 ; -0.38)	-0.50(-0.55 ; -0.45)
16	Were you involved as much as you wanted to be in decisions about your care and treatment?	1.72(1.61 ; 1.83)	1.61(1.54 ; 1.69)	>=1	-1.02(-1.06 ; -0.97)	-1.05(-1.09 ; -1.01)
				2	-0.63(-0.66 ; -0.59)	-0.64(-0.68 ; -0.61)
26	After the operation, did a member of staff explain how it had gone in a way you could understand?	1.34(1.21 ; 1.47)	1.53(1.41 ; 1.65)	>=1	-1.18(-1.27 ; -1.09)	-1.13(-1.20 ; -1.06)
				2	-1.02(-1.10 ; -0.94)	-0.99(-1.05 ; -0.92)
49	Did the doctors or nurses give your family or someone close to you all the information they needed to help care for you at home?	0.93(0.87 ; 0.98)	1.01(0.96 ; 1.05)	>=1	-1.54(-1.62 ; -1.46)	-1.45(-1.51 ; -1.38)
				2	0.88(0.83 ; 0.94)	0.84(0.80 ; 0.89)
**Clinical dimension 3: Experience of NHS staff support in hospital and out of hospital**						
18	How easy or difficult has it been for you to contact your Clinical Nurse Specilaist?	1.11(1.03 ; 1.19)	1.04(0.98 ; 1.09)	>=1	-1.52(-1.62 ; -1.43)	-1.59(-1.67 ; -1.51)
				2	-0.91(-0.97 ; -0.85)	-0.94(-1.00 ; -0.88)
19	When you have had important questions to ask your Clinical Nurse Specialist, how often have you got answers you could understand?	1.28(1.19 ; 1.37)	1.09(1.03 ; 1.15)	>=1	-2.18(-2.30 ; -2.05)	-2.42(-2.54 ; -2.30)
				2	-1.08(-1.15 ; -1.02)	-1.20(-1.26 ; -1.13)
20	Did hospital staff give you information about support or self-help groups for people with cancer?	1.71(1.60 ; 1.82)	1.11(1.05 ; 1.16)	>=1	-1.62(-1.69 ; -1.55)	-2.08(-2.17 ; -1.99)
				2	-0.57(-0.61 ; -0.54)	-0.72(-0.76 ; -0.68)
22	Did hospital staff give you information about how to get financial help or any benefits you might be entitled to?	1.32(1.25 ; 1.40)	0.77(0.73 ; 0.81)	>=1	-1.23(-1.28 ; -1.17)	-1.77(-1.87 ; -1.67)
				2	0.00(-0.03 ; 0.03)	0.03(-0.02 ; 0.07)
41	While you were being treated an an outpatient or day case, did you find someone on the hospital staff to talk to about your worries and fears?	1.06(1.00 ; 1.12)	1.29(1.23 ; 1.35)	>=1	-1.52(-1.60 ; -1.44)	-1.31(-1.37 ; -1.26)
				2	-0.17(-0.21 ; -0.13)	-0.12(-0.16 ; -0.09)
**Clinical dimension 4: Experience of NHS staff support in hospital -inpatient only**						
30	If your family or someone else close to you wanted to talk to a doctor, were they able to?	1.39(1.25 ; 1.53)	1.29(1.22 ; 1.36)	>=1	-0.84(-0.91 ; -0.77)	-0.91(-0.97 ; -0.86)
				2	-0.42(-0.47 ; -0.38)	-0.47(-0.51 ; -0.42)
35	During your hospital visit, did you find someone on the hospital staff to talk to about your worries and fears?	2.07(1.79 ; 2.35)	1.32(1.24 ; 1.39)	>=1	-0.56(-0.61 ; -0.51)	-0.71(-0.77 ; -0.66)
				2	0.01(-0.02 ; 0.05)	0.02(-0.03 ; 0.06)
36	Did the hospital staff do everything they could to help control your pain?	1.07(0.96 ; 1.17)	0.81(0.75 ; 0.87)	>=1	-2.07(-2.22 ; -1.91)	-2.58(-2.76 ; -2.40)
				2	-1.04(-1.12 ; -0.95)	-1.29(-1.39 ; -1.20)
Note: higher value indicates better discrimination and difficulty:1 – discrimination and difficulty in overall model						

DRAFT

Supplementary Table 3. Level of agreement of items in overall IRT model.

Question	Number	Item -test correlation	Item-rest correlation	Average interitem covariance	Alpha
5	14295	0.41	0.33	0.14	0.82
7	14295	0.57	0.45	0.13	0.81
9	15967	0.43	0.31	0.14	0.82
11	15967	0.54	0.40	0.13	0.81
13	15967	0.58	0.46	0.13	0.81
14	3341	0.68	0.59	0.13	0.81
16	15967	0.60	0.49	0.12	0.81
26	5082	0.57	0.47	0.13	0.81
18	13961	0.48	0.35	0.13	0.82
19	13961	0.47	0.37	0.14	0.82
20	15967	0.52	0.41	0.13	0.81
22	15967	0.45	0.32	0.13	0.82
41	14399	0.57	0.46	0.13	0.81
30	8627	0.57	0.46	0.13	0.81
35	7123	0.59	0.49	0.13	0.81
36	8627	0.44	0.34	0.13	0.82
49	15967	0.51	0.41	0.13	0.81
CPES questionnaire				0.13	0.82

Cronbach's alpha coefficient for all questions included in the five models. The alpha coefficient for all the questions are good and for the overall CPES questionnaire (0.82). The inter-test and inter-rest correlations for most of the questions were moderate positive.

DRAFT

Supplementary Table 4. Proportions of respondents completing questions included in IRT models.

Question number	Question description	Answer option number	Answer option	Score of answer option	Response	Code	Number surveyed in wave 1 (%)	Number surveyed in wave 2 (%)	Number surveyed in wave 3 (%)	Number surveyed in wave 4 (%)	Number surveyed in wave 5 (%)	Total number of people in all waves
Q5	Beforehand, did you have all the information you needed about your test?	1	Yes	2	Positive	2	2278(91.4)	2765(92.5)	2772(93.2)	2679(93.6)	2681(90.1)	13175(92.2)
		2	Yes, to some extent	0	Negative	0	172(6.9)	174(5.8)	154(5.2)	145(5.1)	187(6.3)	832(5.8)
		3	No, but I would have liked an explanation	0	Neutral	1	42(1.7)	51(1.7)	49(1.7)	39(1.4)	107(3.6)	288(2.0)
		4	I did not need an explanation	1		Total	2492(100.0)	2990(100.0)	2975(100.0)	2863(100.0)	2975(100.0)	14295(100.0)
		5	Don't know / can't remember	1								
			Unknown	1								
Q7	Were the results of the test explained in a way you could understand?	1	Yes, completely	2	Positive	2	1851(74.3)	2294(76.7)	2296(77.2)	2181(76.2)	2261(76.0)	10883(76.1)
		2	Yes, to some extent	0	Negative	0	548(22.0)	609(20.4)	600(20.2)	612(21.4)	604(20.3)	2973(20.8)
		3	No, but I would have liked an explanation	0	Neutral	1	93(3.7)	87(2.9)	79(2.7)	70(2.4)	110(3.7)	439(3.1)
		4	I did not have an explanation but would have liked one	0		Total	2492(100.0)	2990(100.0)	2,975(100.0)	2,863(100.0)	2,975(100.0)	14295(100.0)
		5	I did not need an explanation	1								
		6	Don't know / can't remember	1								
			Unknown	1								
Q9	How do you feel about the way you were told you had cancer?	1	It was done sensitively	2	Positive	2	2225(80.6)	2718(81.7)	2755(82.2)	2631(82.6)	2725(81.6)	13054(81.8)
		2	It should have been done a bit more sensitively	0	Negative	0	474(17.2)	539(16.2)	542(16.2)	520(16.3)	557(16.7)	2632(16.5)
		3	It should have been done a lot more sensitively	0	Neutral	1	62(2.3)	70(2.1)	56(1.7)	34(1.1)	59(1.8)	281(1.8)
			Unknown	1		Total	2761(100.0)	3,327(100.0)	3,353(100.0)	3,185(100.0)	3,341(100.0)	l5967(100.0)
Q11	When you were told you had cancer, were you given written information about the type of cancer you had?	1	Yes, and it was easy to understand	2	Positive	2	1427(51.7)	1,769(53.2)	1,839(54.9)	1,714(53.8)	1,839(55.0)	8588(53.8)
		2	Yes, but it was difficult to understand	0	Negative	0	893(32.3)	1,064(32.0)	984(29.4)	939(29.5)	991(29.7)	4871(30.5)
		3	No, I was not given written information about the type of cancer I had	0	Neutral	1	441(16.0)	494(14.9)	530(15.8)	532(16.7)	511(15.3)	2508(15.7)
		4	I did not need written information	1		Total	2761(100.0)	3,327(100.0)	3,353(100.0)	3,185(100.0)	3,341(100.0)	l5967(100.0)
		5	Don't know / can't remember	1								
			Unknown	1								
Q13	Were the possible side effects of treatment(s) explained to you in a way you could understand?	1	Yes, definitely	2	Positive	2	1,952(70.7)	2,461(74.0)	2,418(72.1)	2334(73.3)	2,351(70.4)	11516(72.1)
		2	Yes, to some extent	0	Negative	0	622(22.5)	680(20.4)	716(21.4)	671(21.1)	820(24.5)	3509(22.0)
		3	No, side effects were not explained	0	Neutral	1	187(6.8)	186(5.6)	219(6.5)	180(5.7)	170(5.1)	942(5.9)
		4	I did not need an explanation	1		Total	2,761(100.0)	3,327(100.0)	3,353(100.0)	3185(100.0)	3,341(100.0)	15967(100.0)
		5	Don't know / can't remember	1								
			Unknown	1								
Q14	Were you offered practical advice and support in dealing with the side effects of your treatment(s)?	1	Yes, definitely	2	Positive	2					2167(64.9)	2167(64.9)
		2	Yes, to some extent	0	Negative	0					994(29.8)	994(29.8)
		3	No, I was not offered any practical advice or support	0	Neutral	1					180(5.4)	180(5.4)
		4	Don't know / can't remember	1		Total					3,341(100.0)	3,341(100.0)
			Unknown	1								

DRAFT

Q16	Were you involved as much as you wanted to be in decisions about your care and treatment?	1	Yes, definitely	2	Positive	2	1445(52.3)	2,311(69.5)	2333(69.6)	2172(68.2)	2,468(73.9)	10729(67.2)
		2	Yes, to some extent	0	Negative	0	547(19.8)	834(25.1)	848(25.3)	841(26.4)	686(20.5)	3756(23.5)
		3	No, but I would like to have been more involved	0	Neutral	1	769(27.9)	182(5.5)	172(5.1)	172(5.4)	187(5.6)	1482(9.3)
		4	Don't know / can't remember	1	Total		2761(100.0)	3,327(100.0)	3353(100.0)	3185(100.0)	3,341(100.0)	15967(100.0)
			Unknown	1								
Q26	After the operation, did a member of staff explain how it had gone in a way you could understand?	1	Yes, completely	2	Positive	2	578(70.4)	722(73.5)	837(74.1)	755(74.2)	844(74.6)	3736(73.5)
		2	Yes, to some extent	0	Negative	0	209(25.5)	224(22.8)	255(22.6)	230(22.6)	271(24.0)	1189(23.4)
		3	No, but I would have liked an explanation	0	Neutral	1	34(4.1)	37(3.8)	37(3.3)	33(3.2)	16(1.4)	157(3.1)
		4	I did not need an explanation	1	Total		821(100.0)	983(100.0)	1,129(100.0)	1,018(100.0)	1,131(100.0)	5082(100.0)
			Unknown	1								
Q49	Did the doctors or nurses give your family or someone close to you all the information they needed to help care for you at home?	1	Yes, definitely	2	Positive	2	793(28.7)	1011(30.4)	1012(30.2)	918(28.8)	1594(47.7)	5328(33.4)
		2	Yes, to some extent	0	Negative	0	549(19.9)	612(18.4)	644(19.2)	615(19.3)	1205(36.1)	3625(22.7)
		3	No	0	Neutral	1	1,419(51.4)	1,704(51.2)	1,697(50.6)	1652(51.9)	542(16.2)	7014(43.9)
		4	No family or friends were involved	1	Total		2,761(100.0)	3,327(100.0)	3,353(100.0)	3185(100.0)	3341(100.0)	15967(100.0)
		5	My family or friends did not want or need information	1								
		6	I did not want my family or friends to be involved	1								
			Unknown	1								
Q18	How easy or difficult has it been for you to contact your Clinical Nurse Specialist?	1	Easy	2	Positive	2	1,610(67.9)	2,015(69.5)	1,990(68.1)	1,885(67.3)	2,314(78.0)	9814(70.3)
		2	Sometimes easy, sometimes difficult	0	Negative	0	528(22.3)	599(20.7)	654(22.4)	600(21.4)	310(10.5)	2691(19.3)
		3	Difficult	0	Neutral	1	233(9.8)	284(9.8)	280(9.6)	318(11.3)	341(11.5)	1456(10.4)
		4	I have not tried to contact my Clinical Nurse Specialist	1	Total		2,371(100.0)	2,898(100.0)	2,924(100.0)	2,803(100.0)	2,965(100.0)	13961(100.0)
			Unknown	1								
Q19	When you have had important questions to ask your Clinical Nurse Specialist, how often have you got answers you could understand?	1	All of the time	2	Positive	2	1801(76.0)	2248(77.6)	2245(76.8)	2132(76.1)	2160(72.9)	10586(75.8)
		2	Some of the time	0	Negative	0	230(9.7)	251(8.7)	251(8.6)	252(9.0)	281(9.5)	1265(9.1)
		3	Rarely or never	0	Neutral	1	340(14.3)	399(13.8)	428(14.6)	419(15.0)	524(17.7)	2110(15.1)
		4	I have not asked any questions	1	Total		2371(100.0)	2898(100.0)	2,924(100.0)	2803(100.0)	2,965(100.0)	13961(100.0)
			Unknown	1								
Q20	Did hospital staff give you information about support or self-help groups for people with cancer?	1	Yes	2	Positive	2	1802(65.3)	2251(67.7)	2272(67.8)	2090(65.6)	2125(63.6)	10540(66.0)
		2	No, but I would have liked information	0	Negative	0	366(13.3)	394(11.8)	398(11.9)	376(11.8)	474(14.2)	2008(12.6)
		3	It was not necessary	1	Neutral	1	593(21.5)	682(20.5)	683(20.4)	719(22.6)	742(22.2)	3419(21.4)
		4	Don't know / can't remember	1	Total		2,761(100.0)	3327(100.0)	3,353(100.0)	3,185(100.0)	2241(100.0)	15967(100.0)
			Unknown	1								
Q22	Did hospital staff give you information about how to get financial help or any benefits you might be entitled to?	1	Yes	2	Positive	2	1410(51.1)	1796(54.0)	1674(49.9)	1577(49.5)	1530(45.8)	7987(50.0)
		2	No, but I would have liked information	0	Negative	0	628(22.8)	746(22.4)	713(21.3)	679(21.3)	809(24.2)	3575(22.4)
		3	It was not necessary	1	Neutral	1	723(26.2)	785(23.6)	966(28.8)	929(29.2)	1002(30.0)	4405(27.6)
		4	Don't know / can't remember	1	Total		2,761(100.0)	3327(100.0)	3,353(100.0)	3,185(100.0)	3341(100.0)	15967(100.0)

DRAFT

			Unknown	1								
Q41	While you were being treated as an outpatient or day case, did you find someone on the hospital staff to talk to about your worries and fears?	1	Yes, definitely	2	Positive	2	1392(55.6)	1,708(55.9)	1,592(51.7)	1445(49.5)	1,649(58.1)	7786(54.1)
		2	Yes, to some extent	0	Negative	0	487(19.4)	544(17.8)	590(19.2)	599(20.5)	743(26.2)	2963(20.6)
		3	No	0	Neutral	1	627(25.0)	804(26.3)	896(29.1)	875(30.0)	448(15.8)	3650(25.4)
		4	I had no worries or fears	1		Total	2506(100.0)	3,056(100.0)	3,078(100.0)	2919(100.0)	2,840(100.0)	14399(100.0)
			Unknown	1								
Q28	Did groups of doctors and nurses talk in front of you as if you weren't there?	1	Yes, often	0	Positive	2	1226(81.5)	1419(79.5)	1427(78.7)	1377(79.4)	1340(74.9)	6789(78.7)
		2	Yes, sometimes	0	Negative	0	270(18.0)	353(19.8)	364(20.1)	346(20.0)	437(24.4)	1770(20.5)
		3	No	2	Neutral	1	8(0.5)	14(0.8)	22(1.2)	11(0.6)	13(0.7)	68(0.8)
			Unknown	1		Total	1,504(100.0)	1786(100.0)	1813(100.0)	1,734(100.0)	1790(100.0)	8627(100.0)
Q29	Did you have confidence and trust in the doctors treating you?	1	Yes, in all of them	2	Positive	2	1221(81.2)	1471(82.4)	1494(82.4)	1429(82.4)	1447(80.8)	7062(81.9)
		2	Yes, in some of them	0	Negative	0	272(18.1)	304(17.0)	301(16.6)	297(17.1)	329(18.4)	1503(17.4)
		3	No, in none of them	0	Neutral	1	11(0.7)	11(0.6)	18(1.0)	8(0.5)	14(0.8)	62(0.7)
			Unknown	1		Total	1,504(100.0)	1786(100.0)	1,813(100.0)	1,734(100.0)	1,790(100.0)	8627(100.0)
Q30	If your family or someone else close to you wanted to talk to a doctor, were they able to?	1	Yes, definitely	2	Positive	2	907(60.3)	1082(60.6)	1079(59.5)	1065(61.4)	1102(61.6)	5235(60.7)
		2	Yes, to some extent	0	Negative	0	477(31.7)	548(30.7)	559(30.8)	506(29.2)	465(26.0)	2555(29.6)
		3	No	0	Neutral	1	120(8.0)	156(8.7)	175(9.7)	163(9.4)	223(12.5)	837(9.7)
		4	No family or friends were involved	1		Total	1,504(100.0)	1786(100.0)	1,813(100.0)	1,734(100.0)	1790(100.0)	8627(100.0)
		5	My family did not want to talk to a doctor	1								
		6	I did not want my family or friends to talk to a doctor	1								
			Unknown	1								
Q35	During your hospital visit, did you find someone on the hospital staff to talk to about your worries and fears?	1	Yes, definitely	2	Positive	2		991(55.5)	983(54.2)	942(54.3)	637(35.6)	3553(49.9)
		2	Yes, to some extent	0	Negative	0		550(30.8)	560(30.9)	534(30.8)	711(39.7)	2355(33.1)
		3	No	0	Neutral	1		245(13.7)	270(14.9)	258(14.9)	442(24.7)	1215(17.1)
		4	I had no worries or fears	1		Total		1786(100.0)	1,813(100.0)	1,734(100.0)	1,790(100.0)	7123(100.0)
			unknown	1								
Q36	Did the hospital staff do everything they could to help control your pain?	1	Yes, definitely	2	Positive	2	1029(68.4)	1276(71.4)	1317(72.6)	1255(72.4)	1303(72.8)	6180(71.6)
		2	Yes, to some extent	0	Negative	0	190(12.6)	254(14.2)	230(12.7)	219(12.6)	269(15.0)	1162(13.5)
		3	No	0	Neutral	1	285(19.0)	256(14.3)	266(14.7)	260(15.0)	218(12.2)	1285(14.9)
		4	I did not have any pain	1		Total	1,504(100.0)	1786(100.0)	1,813(100.0)	1,734(100.0)	1,790(100.0)	8627(100.0)
			Unknown	1								

DRAFT

Supplementary Table 5. Associations of lung cancer patient characteristics and latent experience of clinical survey sections.

Characteristic	Total number of people (%)		Clinical section 1			Clinical section 2			Clinical section 3	
		Mean(SD)	coefficient(95%CI)	coefficient(95%CI)[a]	Mean(SD)	coefficient(95%CI)	coefficient(95%CI)[a]	Mean(SD)	coefficient(95%CI)	coefficient(95%CI)[a]
**CPES Wave**										
1	3280(20.5)	-0.04(0.70)	0.00	0.00	-0.08(0.68)	0.00	0.00	0.01(0.77)	0.00	0.00
2	3212(20.1)	0.00(0.70)	0.04(0.01 ; 0.08)	0.03(0.00 ; 0.07)	0.01(0.72)	0.10(0.06 ; 0.13)	0.08(0.05 ; 0.12)	0.03(0.78)	0.01(-0.02 ; 0.05)	0.02(-0.02 ; 0.06)
3	3302(20.7)	0.02(0.69)	0.06(0.03 ; 0.10)	0.05(0.01 ; 0.09)	0.01(0.73)	0.09(0.05 ; 0.12)	0.07(0.03 ; 0.11)	0.01(0.75)	0.00(-0.04 ; 0.03)	0.02(-0.02 ; 0.05)
4	3028(19.0)	0.02(0.68)	0.06(0.02 ; 0.09)	0.05(0.01 ; 0.08)	0.00(0.73)	0.08(0.04 ; 0.12)	0.07(0.03 ; 0.10)	-0.03(0.75)	-0.04(-0.08 ; -0.01)	-0.03(-0.07 ; 0.01)
5	3145(19.7)	0.00(0.72)	0.05(0.01 ; 0.08)	0.03(-0.01 ; 0.07)	0.06(0.87)	0.14(0.10 ; 0.17)	0.13(0.08 ; 0.17)	-0.02(0.79)	-0.04(-0.07 ; 0.00)	0.00(-0.04 ; 0.04)
**Sex**										
Male	8561(53.6)	0.02(0.69)	0.00	0.00	0.04(0.74)	0.00	0.00	0.05(0.76)	0.00	0.00
Female	7406(46.4)	-0.03(0.70)	-0.05(-0.07; -0.03)	-0.05(-0.07; -0.03)	-0.04(0.76)	-0.08(-0.10; -0.06)	-0.08(-0.10 ; -0.06)	-0.05(0.78)	-0.10(-0.13; -0.08)	-0.10(-0.12 ; -0.08)
**Age (years)**										
<65	5442(34.1)	-0.05(0.72)	0.00	0.00	-0.03(0.77)	0.00	0.00	0.01(0.81)	0.00	
65-80	9394(58.8)	0.03(0.69)	0.08(0.06 ; 0.11)	0.08(0.05 ; 0.10)	0.02(0.74)	0.05(0.02 ; 0.07)	0.04(0.02 ; 0.07)	0.01(0.75)	0.00(-0.03 ; 0.02)	0.02(-0.01 ; 0.05)
>80	1131(7.1)	-0.01(0.69)	0.05(0.00 ; 0.09)	0.07(0.02 ; 0.11)	-0.02(0.72)	0.00(-0.04 ; 0.05)	0.04(-0.01 ; 0.09)	-0.14(0.71)	-0.16(-0.20; -0.11)	-0.08(-0.12 ; -0.03)
**Ethnicity**										
White	15405(96.5)	0.00(0.70)	0.00	0.00	0.00(0.75)	0.00	0.00	0.00(0.77)	0.00	0.00
Non-white	562(3.5)	-0.08(0.74)	-0.09(-0.15; -0.03)	-0.07(-0.13; -0.01)	-0.10(0.79)	-0.10(-0.17; -0.04)	-0.11(-0.18 ; -0.04)	-0.13(0.83)	-0.14(-0.20; -0.07)	-0.14(-0.21 ; -0.07)
**Stage**										
Stage IA-IB	2884(18.1)	0.04(0.66)	0.00	0.00	-0.03(0.79)	0.00	0.00	-0.31(0.71)	0.00	0.00
Stage IIA-IIB	1930(12.1)	0.04(0.67)	0.01(-0.03 ; 0.04)	0.00(-0.04 ; 0.04)	0.05(0.78)	0.08(0.04 ; 0.13)	0.07(0.02 ; 0.11)	-0.12(0.77)	0.19(0.15 ; 0.24)	0.14(0.10 ; 0.19)
Stage IIIA-IIIB	4547(28.5)	0.02(0.70)	-0.01(-0.04 ; 0.02)	-0.03(-0.06 ; 0.01)	0.01(0.74)	0.04(0.00 ; 0.07)	0.01(-0.03 ; 0.06)	0.06(0.77)	0.36(0.33 ; 0.40)	0.16(0.11 ; 0.20)
Stage IV	4843(30.3)	-0.05(0.72)	-0.08(-0.11; -0.05)	-0.08(-0.12; -0.04)	0.02(0.74)	0.05(0.01 ; 0.08)	0.02(-0.02 ; 0.07)	0.15(0.75)	0.46(0.43 ; 0.49)	0.20(0.16 ; 0.25)
Unknown	1763(11.0)	-0.04(0.71)	-0.08(-0.12; -0.04)	-0.06(-0.11; -0.01)	-0.07(0.69)	-0.04(-0.08 ; 0.01)	0.00(-0.05 ; 0.05)	0.07(0.76)	0.38(0.33 ; 0.42)	0.17(0.12 ; 0.22)
**Deprivation**										
1-least deprived	2557(16.0)	-0.01(0.69)	0.00	0.00	-0.02(0.76)	0.00	0.00	-0.03(0.74)	0.00	0.00
2	3222(20.2)	-0.02(0.70)	0.00(-0.04 ; 0.03)	-0.01(-0.04 ; 0.03)	-0.05(0.76)	-0.03(-0.07 ; 0.01)	-0.03(-0.07 ; 0.01)	-0.02(0.75)	0.01(-0.03 ; 0.05)	0.01(-0.03 ; 0.04)
3	3297(20.7)	0.00(0.69)	0.02(-0.02 ; 0.05)	0.02(-0.02 ; 0.05)	0.00(0.74)	0.02(-0.02 ; 0.06)	0.03(-0.01 ; 0.06)	-0.02(0.77)	0.00(-0.03 ; 0.04)	0.01(-0.03 ; 0.05)
4	3354(21.0)	0.02(0.70)	0.03(-0.01 ; 0.06)	0.03(-0.01 ; 0.07)	0.02(0.74)	0.04(0.01 ; 0.08)	0.05(0.01 ; 0.09)	0.01(0.79)	0.04(0.00 ; 0.08)	0.04(0.01 ; 0.08)
5-most deprived	3537(22.2)	0.00(0.71)	0.02(-0.02 ; 0.05)	0.02(-0.02 ; 0.06)	0.03(0.75)	0.05(0.01 ; 0.09)	0.06(0.02 ; 0.10)	0.04(0.79)	0.07(0.03 ; 0.11)	0.08(0.04 ; 0.12)
**Comorbidity score**										
0	6589(41.3)	0.01(0.69)	0.00	0.00	0.00(0.74)	0.00	0.00	0.02(0.77)	0.00	0.00
1	3473(21.8)	0.01(0.69)	0.00(-0.03 ; 0.03)	-0.01(-0.04 ; 0.02)	-0.01(0.75)	-0.01(-0.04 - 0.02)	-0.01(-0.05 ; 0.02)	-0.02(0.77)	-0.05(-0.08; -0.01)	-0.02(-0.05 ; 0.01)
2-3	2428(15.2)	0.04(0.69)	0.03(0.00 ; 0.06)	0.02(-0.02 ; 0.05)	0.04(0.75)	0.04(0.00 - 0.07)	0.03(0.00 ; 0.07)	-0.07(0.76)	-0.10(-0.13; -0.06)	-0.02(-0.05 ; 0.02)
4+	3477(21.8)	-0.05(0.72)	-0.06(-0.09; -0.03)	-0.04(-0.07; -0.01)	-0.02(0.77)	-0.02(-0.05 - 0.01)	-0.02(-0.05 ; 0.01)	0.03(0.78)	0.01(-0.02 ; 0.04)	-0.02(-0.05 ; 0.01)
**Route of diagnosis**										
GP referral	4062(25.4)	-0.01(0.70)	0.00	0.00	-0.02(0.76)	0.00	0.00	-0.06(0.78)	0.00	0.00
Emergency presentation	2012(12.6)	-0.11(0.76)	-0.11(-0.15; -0.07)	-0.08(-0.12; -0.04)	-0.03(0.78)	-0.01(-0.05 ; 0.03)	0.00(-0.05 ; 0.04)	0.01(0.80)	0.07(0.03 ; 0.11)	-0.01(-0.05 ; 0.04)

DRAFT

Inpatient elective	287(1.8)	0.10(0.62)	0.11(0.03 ; 0.18)	0.12(0.05 ; 0.20)	0.11(0.71)	0.14(0.05 ; 0.23)	0.14(0.05 ; 0.22)	0.16(0.78)	0.22(0.13 ; 0.31)	0.12(0.03 ; 0.21)
Other outpatient	2161(13.5)	0.00(0.68)	0.01(-0.03 ; 0.04)	0.00(-0.03 ; 0.04)	-0.01(0.75)	0.01(-0.02 ; 0.05)	0.01(-0.03 ; 0.05)	-0.06(0.76)	-0.01(-0.05 ; 0.03)	0.02(-0.02 ; 0.06)
TWW	7318(45.8)	0.03(0.68)	0.04(0.01 ; 0.06)	0.03(0.00 ; 0.06)	0.02(0.73)	0.05(0.02 ; 0.08)	0.03(0.00 ; 0.06)	0.04(0.75)	0.10(0.07 ; 0.13)	0.04(0.01 ; 0.07)
Missing	127(0.8)	-0.11(0.67)	-0.10(-0.22 ; 0.02)	-0.07(-0.19 ; 0.04)	-0.10(0.75)	-0.07(-0.21 ; 0.06)	-0.05(-0.18 ; 0.08)	-0.12(0.74)	-0.06(-0.19 ; 0.07)	-0.07(-0.20 ; 0.05)
**Lung Cancer type**										
NSCLC	13,268(83.1)	-0.01(0.70)	0.00	0.00	-0.01(0.75)	0.00	0.00	-0.03(0.77)	0.00	0.00
Carcinoid	273(1.7)	-0.06(0.66)	-0.05(-0.13 ; 0.02)	-0.05(-0.13 ; 0.03)	-0.13(0.78)	-0.12(-0.21; -0.03)	-0.07(-0.16 ; 0.03)	-0.40(0.64)	-0.37(-0.45 ; -0.29)	-0.14(-0.22 ; -0.06)
SCLC	2,426(15.2)	0.04(0.70)	0.05(0.02 ; 0.08)	0.06(0.03 ; 0.10)	0.05(0.72)	0.06(0.03 ; 0.09)	0.05(0.02 ; 0.08)	0.19(0.74)	0.22(0.19 ; 0.25)	0.08(0.05 ; 0.12)
**Received anti-cancer treatment**										
Surgery	5272(33.0)	0.03(0.66)	0.00	0.00	-0.01(0.81)	0.00	0.00	-0.24(0.74)	0.00	0.00
Other	10695(67.0)	-0.01(0.71)	-0.04(-0.06; -0.02)	-0.02(-0.05 ; 0.02)	0.00(0.72)	0.01(-0.01 ; 0.04)	-0.01(-0.04 ; 0.03)	0.12(0.76)	0.36(0.34 ; 0.39)	0.23(0.19 ; 0.26)
**Number of anti-cancer treatment**										
0	537(3.4)	-0.21(0.73)	0.00	0.00	-0.27(0.69)	0.00	0.00	-0.20(0.76)	0.00	0.00
1	7,216(45.2)	0.00(0.69)	0.20(0.14 ; 0.26)	0.18(0.11 ; 0.24)	-0.01(0.75)	0.25(0.19 ; 0.32)	0.24(0.18 ; 0.31)	-0.07(0.76)	0.13(0.07 ; 0.20)	0.16(0.09 ; 0.23)
2	7,727(48.4)	0.02(0.69)	0.22(0.16 ; 0.29)	0.20(0.13 ; 0.26)	0.03(0.75)	0.29(0.23 ; 0.36)	0.27(0.21 ; 0.34)	0.08(0.76)	0.28(0.22 ; 0.35)	0.21(0.14 ; 0.27)
3	487(3.1)	-0.01(0.72)	0.19(0.10 ; 0.28)	0.15(0.06 ; 0.24)	0.00(0.83)	0.26(0.17 ; 0.35)	0.24(0.14 ; 0.34)	-0.06(0.78)	0.15(0.05 ; 0.24)	0.15(0.05 ; 0.25)
**Anti-cancer treatment modality**										
Surgery	5,272(33.0)	0.03(0.66)	0.00	0.00	-0.01(0.81)	0.00	0.00	-0.24(0.74)	0.00	0.00
No treatment	537(3.4)	-0.21(0.73)	-0.23(-0.29 ; -0.17)	-0.19(-0.25; -0.12)	-0.27(0.69)	-0.26(-0.32; -0.19)	-0.24(-0.31 ; -0.18)	-0.20(0.76)	0.04(-0.03 ; 0.11)	0.00(-0.07 ; 0.07)
Chemo and radio	5,680(35.6)	0.01(0.70)	-0.02(-0.04 ; 0.01)	0.01(-0.02 ; 0.05)	0.02(0.72)	0.03(0.00 ; 0.06)	0.02(-0.02 ; 0.06)	0.15(0.75)	0.40(0.37 ; 0.42)	0.27(0.23 ; 0.31)
Chemotherapy alone	3,085(19.3)	-0.03(0.72)	-0.05(-0.08; -0.02)	0.00(-0.04 ; 0.04)	0.03(0.73)	0.04(0.00 ; 0.07)	0.04(0.00 ; 0.08)	0.18(0.74)	0.42(0.39 ; 0.46)	0.31(0.27 ; 0.35)
Radiotherapy only	1,393(8.7)	-0.02(0.72)	-0.05(-0.09; -0.01)	-0.03(-0.07 ; 0.01)	-0.03(0.71)	-0.03(-0.07 ; 0.02)	-0.03(-0.07 ; 0.02)	-0.04(0.78)	0.20(0.16 ; 0.24)	0.15(0.10 ; 0.20)
**Anti-cancer treatment modality (NSCLC)**										
Surgery	4,945(37.3)	0.03(0.66)	0.00	0.00	0.00(0.81)	0.00	0.00	-0.24(0.74)	0.00	0.00
No treatment	496(3.7)	-0.22(0.73)	-0.25(-0.31 ; -0.18)	-0.20(-0.27; -0.13)	-0.28(0.69)	-0.28(-0.35; -0.21)	-0.26(-0.33 ; -0.19)	-0.21(0.76)	0.03(-0.04 ; 0.10)	0.00(-0.07 ; 0.07)
Chemo and radio	3,750(28.3)	-0.01(0.71)	-0.05(-0.08; -0.02)	0.00(-0.04 ; 0.04)	0.00(0.73)	0.00(-0.03 ; 0.04)	0.02(-0.03 ; 0.06)	0.12(0.75)	0.36(0.33 ; 0.39)	0.26(0.22 ; 0.30)
Chemotherapy alone	2,769(20.9)	-0.02(0.72)	-0.06(-0.09; -0.02)	0.01(-0.03 ; 0.05)	0.03(0.72)	0.03(0.00 ; 0.07)	0.05(0.00 ; 0.09)	0.18(0.73)	0.42(0.39 ; 0.45)	0.32(0.27 ; 0.36)
Radiotherapy only	1,308(9.9)	-0.02(0.72)	-0.05(-0.09; -0.01)	-0.03(-0.08 ; 0.01)	-0.03(0.71)	-0.03(-0.07 ; 0.02)	-0.03(-0.07 ; 0.02)	-0.05(0.78)	0.19(0.14 ; 0.23)	0.15(0.10 ; 0.21)
**Anti-cancer treatment modality (SCLC)**										
Surgery	102(4.2)	0.00(0.73)	0.00	0.00	-0.01(0.90)	0.00	0.00	-0.01(0.74)	0.00	0.00
No treatment	21(0.9)	-0.10(0.79)	-0.10(-0.43 ; 0.23)	-0.07(-0.45 - 0.31)	-0.14(0.70)	-0.13(-0.47 ; 0.21)	-0.15(-0.51 ; 0.21)	0.16(0.73)	0.16(-0.18 ; 0.51)	0.13(-0.24 ; 0.49)
Chemo and radio	1,921(79.2)	0.06(0.69)	0.06(-0.08 ; 0.20)	0.09(-0.08 - 0.26)	0.07(0.70)	0.07(-0.07 ; 0.22)	0.05(-0.15 ; 0.25)	0.21(0.74)	0.22(0.07 ; 0.37)	0.18(-0.01 ; 0.36)
Chemotherapy alone	302(12.5)	-0.03(0.73)	-0.03(-0.18 ; 0.13)	0.00(-0.18 - 0.19)	0.03(0.74)	0.04(-0.12 ; 0.20)	0.00(-0.22 ; 0.21)	0.17(0.76)	0.18(0.01 ; 0.35)	0.11(-0.09 ; 0.32)
Radiotherapy only	80(3.3)	-0.03(0.76)	-0.03(-0.23 ; 0.18)	0.02(-0.22 - 0.26)	-0.09(0.71)	-0.08(-0.29 ; 0.13)	-0.06(-0.31 ; 0.20)	0.06(0.77)	0.07(-0.15 ; 0.29)	0.03(-0.21 ; 0.28)

		Clinical section 4			Overall experience of care pathway		
**Characteristic**	**Total number of people (%)**	**Mean(SD)**	**coefficient(95%CI)**	**coefficient(95%CI)[a]**	**Mean(SD)**	**coefficient(95%CI)**	**coefficient(95%CI)[a]**
**Year of diagnosis**							
2009/2010	3280(20.5)	-0.02(0.45)	0.00	0.00	-0.06(0.82)	0.00	0.00

DRAFT

2011	3212(20.1)	0.02(0.56)	0.04(0.01 ; 0.06)	0.03(0.00 ; 0.05)	0.03(0.87)	0.08(0.04 ; 0.13)	0.07(0.03 ; 0.12)
2012	3302(20.7)	0.02(0.56)	0.04(0.02 ; 0.07)	0.03(0.00 ; 0.05)	0.02(0.86)	0.08(0.04 ; 0.12)	0.07(0.02 ; 0.11)
2013	3028(19.0)	0.03(0.55)	0.04(0.02 ; 0.07)	0.03(0.00 ; 0.06)	-0.01(0.85)	0.05(0.01 ; 0.09)	0.04(0.00 ; 0.09)
2014/2015	3145(19.7)	-0.06(0.54)	-0.04(-0.06; -0.01)	-0.05(-0.08; -0.03)	0.02(0.94)	0.08(0.03 ; 0.12)	0.07(0.03 ; 0.12)
**Sex**							
Male	8561(53.6)	0.03(0.52)	0.00	0.00	0.06(0.86)	0.00	0.00
Female	7406(46.4)	-0.03(0.55)	-0.06(-0.08; -0.04)	-0.06(-0.07; -0.04)	-0.06(0.88)	-0.12(-0.15; -0.09)	-0.12(-0.14 ; -0.09)
**Age (years)**							
<65	5442(34.1)	-0.03(0.58)	0.00	0.00	-0.04(0.92)	0.00	0.00
65-80	9394(58.8)	0.02(0.52)	0.04(0.03 ; 0.06)	0.04(0.02 ; 0.06)	0.03(0.85)	0.07(0.04 ; 0.10)	0.08(0.05 ; 0.11)
>80	1131(7.1)	0.00(0.43)	0.03(0.00 ; 0.06)	0.03(0.00 ; 0.06)	-0.08(0.80)	-0.04(-0.09 ; 0.01)	0.02(-0.03 ; 0.07)
**Ethnicity**							
White	15405(96.5)	0.00(0.53)	0.00	0.00	0.01(0.87)	0.00	0.00
Other	562(3.5)	-0.04(0.54)	-0.04(-0.08 ; 0.01)	-0.03(-0.07 ; 0.02)	-0.15(0.92)	-0.15(-0.23; -0.07)	-0.15(-0.23 ; -0.07)
**Stage**							
Stage IA-IB	2884(18.1)	0.04(0.62)	0.00	0.00	-0.11(0.85)	0.00	0.00
Stage IIA-IIB	1930(12.1)	0.03(0.60)	-0.01(-0.05 ; 0.02)	-0.02(-0.05 ; 0.02)	0.01(0.88)	0.12(0.07 ; 0.17)	0.09(0.04 ; 0.14)
Stage IIIA-IIIB	4547(28.5)	0.00(0.50)	-0.04(-0.07; -0.02)	-0.03(-0.06 ; 0.00)	0.04(0.88)	0.15(0.11 ; 0.19)	0.05(0.00 ; 0.10)
Stage IV	4843(30.3)	-0.03(0.49)	-0.07(-0.10; -0.05)	-0.04(-0.08; -0.01)	0.04(0.88)	0.15(0.11 ; 0.19)	0.05(-0.01 ; 0.10)
Unknown	1763(11.0)	-0.03(0.48)	-0.07(-0.11; -0.04)	-0.05(-0.09; -0.01)	-0.04(0.83)	0.07(0.02 ; 0.12)	0.03(-0.03 ; 0.09)
**Deprivation**							
1-least deprived	2557(16.0)	-0.02(0.53)	0.00	0.00	-0.04(0.84)	0.00	0.00
2	3222(20.2)	-0.01(0.53)	0.01(-0.02 ; 0.04)	0.01(-0.02 ; 0.04)	-0.04(0.87)	0.00(-0.05 ; 0.04)	0.00(-0.05 ; 0.04)
3	3297(20.7)	-0.01(0.53)	0.01(-0.02 ; 0.03)	0.01(-0.02 ; 0.03)	-0.01(0.86)	0.02(-0.02 ; 0.07)	0.03(-0.01 ; 0.07)
4	3354(21.0)	0.00(0.53)	0.02(-0.01 ; 0.05)	0.02(-0.01 ; 0.05)	0.03(0.88)	0.07(0.02 ; 0.11)	0.07(0.03 ; 0.12)
5-most deprived	3537(22.2)	0.02(0.54)	0.04(0.01 ; 0.06)	0.04(0.01 ; 0.07)	0.05(0.89)	0.09(0.05 ; 0.13)	0.10(0.05 ; 0.14)
**Charlson index**							
0	6589(41.3)	-0.01(0.52)	0.00	0.00	0.01(0.87)	0.00	0.00
1	3473(21.8)	0.02(0.52)	0.03(0.01 ; 0.05)	0.02(-0.01 ; 0.04)	0.00(0.86)	-0.01(-0.05 ; 0.02)	-0.01(-0.05 ; 0.02)
2-3	2428(15.2)	0.03(0.55)	0.04(0.02 ; 0.07)	0.02(-0.01 ; 0.05)	0.01(0.86)	0.01(-0.03 ; 0.05)	0.02(-0.02 ; 0.06)
4+	3477(21.8)	-0.02(0.55)	0.00(-0.02 ; 0.02)	0.00(-0.02 ; 0.02)	-0.02(0.89)	-0.03(-0.07 ; 0.00)	-0.04(-0.07 ; 0.00)
**Route of diagnosis**							
GP referral	4062(25.4)	0.00(0.54)	0.00	0.00	-0.04(0.88)	0.00	0.00
Emergency presentation	2012(12.6)	-0.03(0.57)	-0.04(-0.07; -0.01)	-0.02(-0.05 ; 0.01)	-0.07(0.93)	-0.03(-0.08 ; 0.02)	-0.04(-0.09 ; 0.01)
Inpatient elective	287(1.8)	-0.02(0.53)	-0.02(-0.09 ; 0.04)	-0.01(-0.07 ; 0.06)	0.16(0.88)	0.20(0.10 ; 0.31)	0.17(0.07 ; 0.28)
Other outpatient	2161(13.5)	0.02(0.56)	0.01(-0.01 ; 0.04)	0.01(-0.02 ; 0.04)	-0.02(0.86)	0.01(-0.03 ; 0.06)	0.02(-0.03 ; 0.06)
TWW	7318(45.8)	0.00(0.51)	0.00(-0.02 ; 0.02)	0.00(-0.02 ; 0.02)	0.04(0.85)	0.08(0.04 ; 0.11)	0.04(0.01 ; 0.08)
Missing	127(0.8)	0.00(0.53)	-0.01(-0.10 ; 0.09)	0.02(-0.08 ; 0.11)	-0.13(0.85)	-0.10(-0.25 ; 0.05)	-0.07(-0.22 ; 0.08)
**Lung Cancer type**							
NSCLC	13,268(83.1)	0.00(0.54)	0.00	0.00	-0.02(0.87)	0.00	0.00
Carcinoid	273(1.7)	-0.06(0.66)	-0.06(-0.14 ; 0.02)	-0.07(-0.15 ; 0.01)	-0.25(0.81)	-0.24(-0.33; -0.14)	-0.12(-0.22 ; -0.02)

DRAFT

SCLC	2,426(15.2)	-0.01(0.49)	-0.01(-0.03 ; 0.01)	0.01(-0.01 ; 0.03)	0.12(0.87)	0.14(0.10 ; 0.17)	0.09(0.05 ; 0.13)
**Received anti-cancer treatment**							
Surgery	5272(33.0)	0.04(0.65)	0.00	0.00	-0.07(0.88)	0.00	0.00
Other	10695(67.0)	-0.02(0.46)	-0.06(-0.08; -0.04)	-0.04(-0.07; -0.02)	0.04(0.86)	0.11(0.08 ; 0.14)	0.06(0.02 ; 0.10)
**Number of anti-cancer treatment**							
0	537(3.4)	-0.03(0.43)	0.00	0.00	-0.32(0.80)	0.00	0.00
1	7,216(45.2)	0.00(0.54)	0.03(-0.01 ; 0.07)	0.02(-0.02 ; 0.06)	-0.04(0.86)	0.29(0.22 ; 0.36)	0.28(0.21 ; 0.35)
2	7,727(48.4)	0.01(0.52)	0.04(0.00 ; 0.08)	0.04(0.00 ; 0.08)	0.06(0.87)	0.38(0.31 ; 0.45)	0.33(0.25 ; 0.40)
3	487(3.1)	-0.03(0.66)	0.00(-0.07 ; 0.07)	-0.01(-0.08 ; 0.06)	-0.03(0.97)	0.30(0.19 ; 0.41)	0.26(0.15 ; 0.37)
**Anti-cancer treatment modality**							
Surgery	5,272(33.0)	0.04(0.65)	0.00	0.00	-0.07(0.88)	0.00	0.00
No treatment	537(3.4)	-0.03(0.43)	-0.07(-0.12; -0.02)	-0.06(-0.10; -0.01)	-0.32(0.80)	-0.25(-0.32; -0.18)	-0.23(-0.31 ; -0.16)
Chemo and radio	5,680(35.6)	-0.01(0.47)	-0.05(-0.07; -0.03)	-0.03(-0.06 ; 0.00)	0.07(0.86)	0.15(0.11 ; 0.18)	0.11(0.06 ; 0.15)
Chemotherapy alone	3,085(19.3)	-0.04(0.48)	-0.07(-0.10; -0.05)	-0.05(-0.08; -0.02)	0.07(0.87)	0.14(0.10 ; 0.18)	0.13(0.08 ; 0.18)
Radiotherapy only	1,393(8.7)	-0.02(0.43)	-0.06(-0.09; -0.03)	-0.06(-0.09; -0.03)	-0.05(0.87)	0.02(-0.03 ; 0.07)	0.01(-0.05 ; 0.06)
**Anti-cancer treatment modality (NSCLC)**							
Surgery	4,945(37.3)	0.04(0.65)	0.00	0.00	-0.06(0.88)	0.00	0.00
No treatment	496(3.7)	-0.03(0.43)	-0.08(-0.13; -0.03)	-0.07(-0.11; -0.02)	-0.34(0.80)	-0.28(-0.35; -0.20)	-0.25(-0.33 ; -0.17)
Chemo and radio	3,750(28.3)	-0.02(0.46)	-0.06(-0.08; -0.04)	-0.03(-0.06 ; 0.00)	0.04(0.86)	0.10(0.07 ; 0.14)	0.10(0.05 ; 0.14)
Chemotherapy alone	2,769(20.9)	-0.03(0.47)	-0.08(-0.10; -0.05)	-0.04(-0.07 ; 0.00)	0.07(0.86)	0.13(0.09 ; 0.17)	0.14(0.09 ; 0.19)
Radiotherapy only	1,308(9.9)	-0.02(0.43)	-0.07(-0.10; -0.03)	-0.06(-0.09; -0.03)	-0.06(0.87)	0.01(-0.05 ; 0.06)	0.01(-0.05 ; 0.06)
**Anti-cancer treatment modality (SCLC)**							
Surgery	102(4.2)	-0.03(0.70)	0.00	0.00	-0.01(1.02)	0.00	0.00
No treatment	21(0.9)	-0.10(0.44)	-0.07(-0.30 ; 0.16)	-0.10(-0.34 ; 0.14)	-0.09(0.77)	-0.08(-0.46 ; 0.30)	-0.09(-0.51 ; 0.33)
Chemo and radio	1,921(79.2)	0.00(0.48)	0.03(-0.06 ; 0.13)	-0.02(-0.18 ; 0.14)	0.14(0.85)	0.16(-0.04 ; 0.36)	0.13(-0.11 ; 0.37)
Chemotherapy alone	302(12.5)	-0.04(0.49)	0.00(-0.11 ; 0.11)	-0.06(-0.23 ; 0.11)	0.07(0.89)	0.08(-0.14 ; 0.31)	0.03(-0.23 ; 0.29)
Radiotherapy only	80(3.3)	0.00(0.42)	0.04(-0.11 ; 0.18)	0.01(-0.17 ; 0.19)	-0.03(0.86)	-0.02(-0.29 ; 0.25)	-0.01(-0.31 ; 0.30)

DRAFT

Supplementary Figure 1. Test Characteristic Curve of overall experience of care pathway

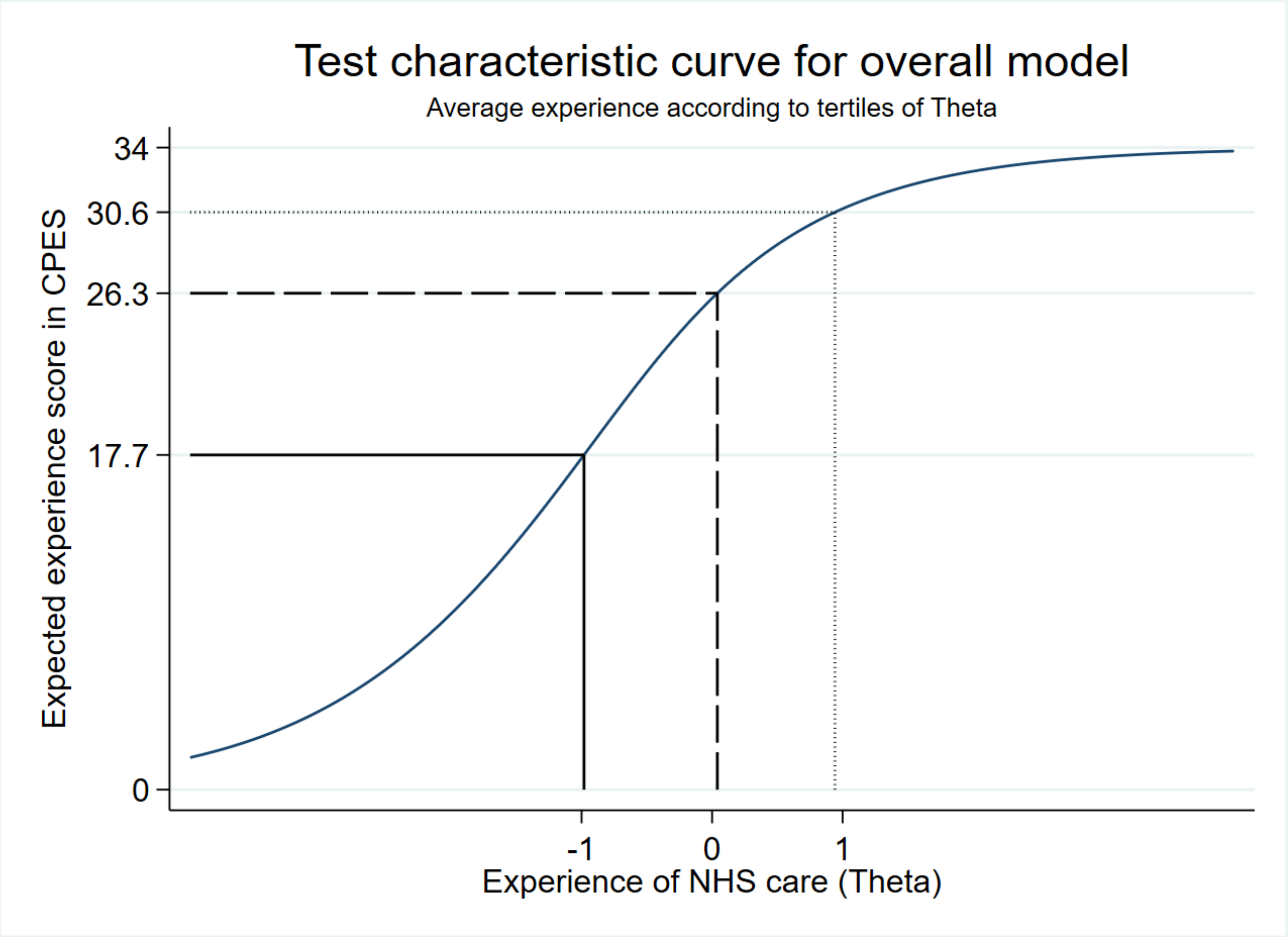

Supplementary Figure 1 legend. Test characteristic curve to demonstrate the expected experience score from 17 CPES questions relative to the theta value of overall experience of cancer care pathway (max score 34; 0-negative, 1-neutral, 2-positive). Mean values of tertiles provide a theta of -1, 0, 1 for negative (solid), average (dash) and positive tertiles (dot), respectively.

DRAFT

Supplementary Figure 2. Boundary characteristic curve for discrimination of items included in IRT models

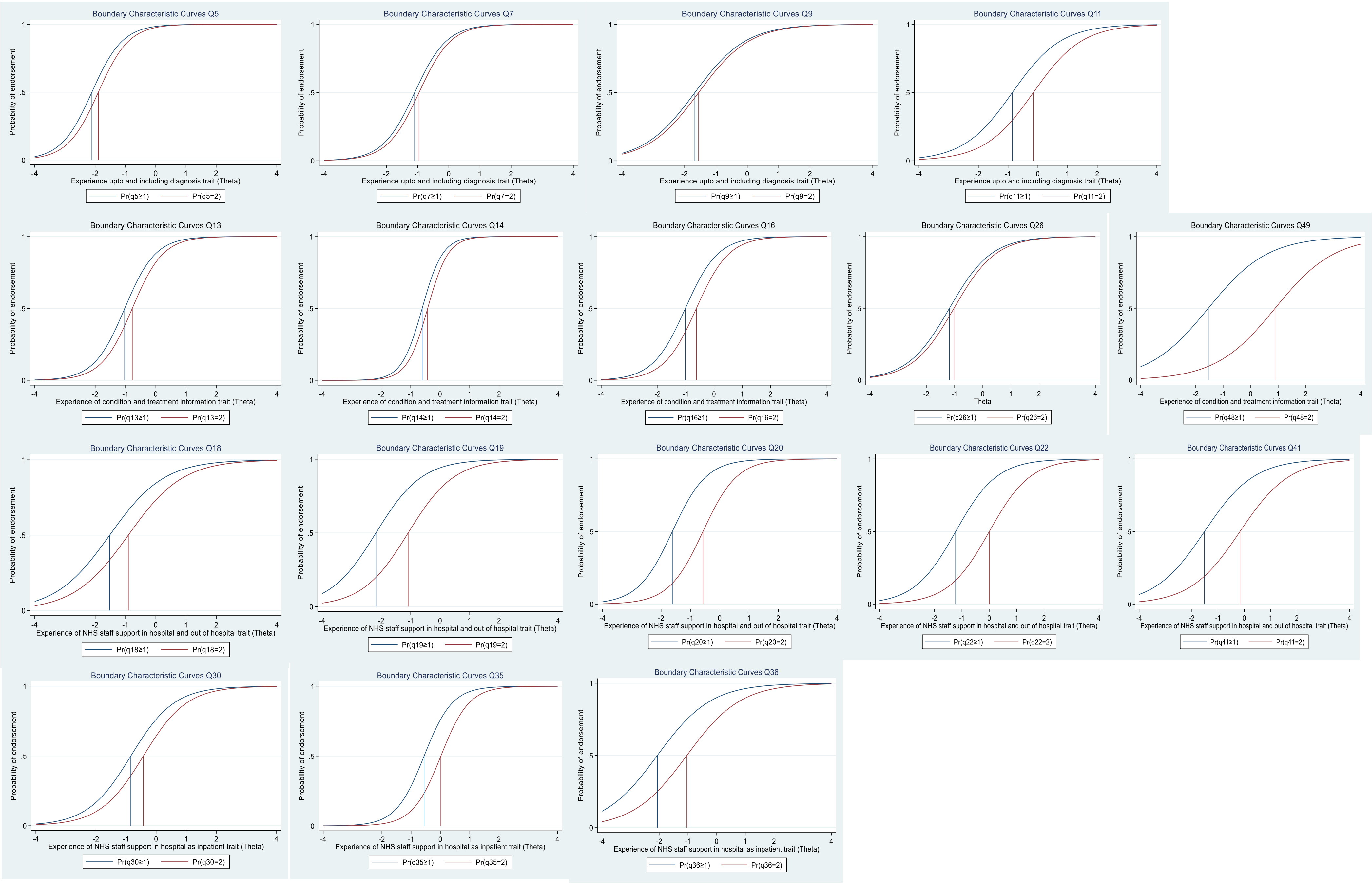

Supplement: Supplementary file 1 — Supplementary file1 (PDF 1986 KB) [file 520_2022_6863_MOESM1_ESM.pdf]
